# Molecular evolution of a chordate specific family of G protein-coupled receptors

**DOI:** 10.1186/1471-2148-11-234

**Published:** 2011-08-09

**Authors:** Stefan Kurtenbach, Christoph Mayer, Thomas Pelz, Hanns Hatt, Florian Leese, Eva M Neuhaus

**Affiliations:** 1 Department of Cell Physiology, Ruhr University Bochum, Universitaetsstrasse 150, 44801 Bochum, Germany; 2 Zoologisches Forschungsmuseum Alexander Koenig, Adenauerallee 160-163, 53113 Bonn, Germany; 3 NeuroScience Research Center, Charité - Universitätsmedizin Berlin, Charitéplatz 1, 10117 Berlin, Germany; 4 Department of Animal Ecology, Evolution and Biodiversity, Ruhr-University Bochum, Universitaetsstrasse 150, 44801 Bochum, Germany; 5 author to whom correspondence should be addressed, Eva M. Neuhaus, NeuroScience Research Center, Charité, Charitéplatz 1, 10117 Berlin, Germany

**Keywords:** GPRC5, chordate development, retinoic acid, phylogeny, GPCR

## Abstract

**Background:**

Chordate evolution is a history of innovations that is marked by physical and behavioral specializations, which led to the development of a variety of forms from a single ancestral group. Among other important characteristics, vertebrates obtained a well developed brain, anterior sensory structures, a closed circulatory system and gills or lungs as blood oxygenation systems. The duplication of pre-existing genes had profound evolutionary implications for the developmental complexity in vertebrates, since mutations modifying the function of a duplicated protein can lead to novel functions, improving the evolutionary success.

**Results:**

We analyzed here the evolution of the GPRC5 family of G protein-coupled receptors by comprehensive similarity searches and found that the receptors are only present in chordates and that the size of the receptor family expanded, likely due to genome duplication events in the early history of vertebrate evolution. We propose that a single GPRC5 receptor coding gene originated in a stem chordate ancestor and gave rise by duplication events to a gene family comprising three receptor types (GPRC5A-C) in vertebrates, and a fourth homologue present only in mammals (GPRC5D). Additional duplications of GPRC5B and GPRC5C sequences occurred in teleost fishes. The finding that the expression patterns of the receptors are evolutionarily conserved indicates an important biological function of these receptors. Moreover, we found that expression of GPRC5B is regulated by vitamin A *in vivo*, confirming previous findings that linked receptor expression to retinoic acid levels in tumor cell lines and strengthening the link between the receptor expression and the development of a complex nervous system in chordates, known to be dependent on retinoic acid signaling.

**Conclusions:**

GPRC5 receptors, a class of G protein-coupled receptors with unique sequence characteristics, may represent a molecular novelty that helped non-chordates to become chordates.

## Background

G protein-coupled receptors (GPCRs) are diverse in their primary structure, which has been used for their phylogenetic classification into subfamilies [1,2]. Most GPCRs belong to one of five subfamilies; Rhodopsin (also termed family A), Adhesion and Secretin (together formerly classified as family B), Glutamate (also termed family C) and Frizzled/Taste2. The Rhodopsin receptor family contains ~670 out of a total of ~800 full-length human receptor proteins [3] and is highly heterogeneous in primary structure and ligand specificity, although most Rhodopsin family receptors do share specific sequence motifs. Family C includes a total of 22 human proteins, metabotropic glutamate receptors, the calcium sensing receptor, γ-aminobutyric acid type B receptors, the sweet and umami taste receptors, GPRC6A and several orphan receptors [4]. While the vast majority of GPCRs have short N-termini and (proposed) agonist binding sites within the seven-transmembrane (7TM) domains, family C receptors are characterized by a large extracellular amino-terminal domain of up to 600 amino acids, which has been shown to function as a ligand binding site [4].

GPRC5 receptors are classified as members of the GPCR family C, but have a shorter N-terminus compared to the other family members. They build a subfamily with four known members GPRC5A, GPRC5B, GPRC5C, and GPRC5D, for which no specific ligands are described [5]. GPRC5A is expressed at high levels in the lung [6-8], whereas the expression pattern of GPRC5B is mostly neuronal with some expression in other tissues [9-11]. GPRC5C is expressed more widely than GPRC5A and GPRC5B, with high expression levels in, e.g., kidney, liver and cerebellum [10]. GPRC5D was found to be associated with hard-keratinized structures, like cortical cells of the hair shaft [12]. The role of these receptors in the tissues they are expressed in is so far unknown. *In vitro *studies showed that expression of GPRC5 receptors can be upregulated by retinoic acid (RA), a metabolite of vitamin A, which lead to their classification as retinoic acid inducible genes (RAIG) [10,13,14]. The finding of transcriptional regulation of GPRC5 receptors by RA is remarkable, because RA is known to play crucial roles in cell differentiation and neurogenesis [15]. The aim of this study was to examine the evolution of GPRC5 receptors. Using similarity searches, we compared GPRC5 receptor sequences in all listed species, analyzed expression patterns and examined their regulation by RA.

## Results

### GPRC5 receptor families

An initial BLAST search for GPRC5 sequences in different species was carried out using the ENSEMBL database (E-value threshold = 10). Similarity searches showed that the four known GPRC5 receptor sequences can be found in all mammalian genomes, while additional sequences sharing higher similarities to GPRC5 receptors than to other GPCRs do not exist in mammals. Interestingly, GPRC5D is only present in mammals, but neither in other tetrapods nor in fish. Birds, reptiles, amphibians, teleost fish and cartilaginous fish have three receptor sequences (GPRC5A-C), *Xenopus *has an additional GPRC5 sequence previously published as XRAIG4 [16]. The genome of the lamprey *Petromyzon marinus*, a jawless vertebrate, contains two GPRC5-like sequences. In addition, we found single GPRC5-like sequences in two urochordates (*Ciona intestinalis *and *Ciona savignyi*) and in the lancelet amphioxus, a cephalochordate species. These sequences likely represent ancestral forms of GPRC5 receptors. Receptor homologues were not found in the genome of *Oikopleura dioica*, another urochordate species belonging to the class of Appendicularia.

**Figure 1:**
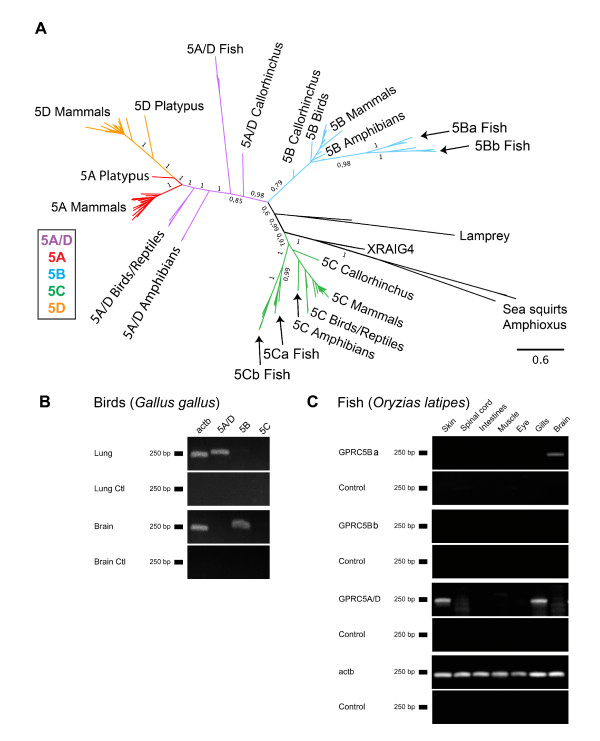
**Phylogenetic analysis and conserved expression of GPRC5 receptors**. (**A**) Phylogenetic tree obtained by Bayesian analysis, branch labels indicate posterior probabilities and ML-bootstrap values. (**B) **Amplification of GPRC5A-C in lung and brain from chicken (*Gallus gallus*) by RT-PCR. Beta-actin (Actb) was used as positive control, mRNA without RT as negative control. (**C**) Amplification of GPRC5A/D, GPRC5Ba and GPRC5Bb in skin, spinal cord, intestines, muscle, eye, gills, lung and brain from fish (*Oryzias latipes*) by RT-PCR. Beta-actin (Actb) was used as positive control.

Prottest determined the JTT+G+F amino acid substitution model as most appropriate for the phylogenetic analysis. Therefore, RAxML analyses were conducted using the PROTGAMMAJTTF model, and for the MrBayes analyses the parameters "aamodelpr = fixed(jones)" and "rates = gamma" (amino acid frequencies could not be varied in MrBayes) were used. The burnin value for the Bayesian analysis of the main data set was 10500. The complete phylogenetic tree of the "main data set" is shown in Figure 1A. The topologies of the Bayesian and the ML tree are largely identical (ML tree shown in Additional file 1, Figure S1A). Posterior probabilities are usually higher than bootstrap support values. Mammalian sequences of the receptor types GPRC5A and GPRC5D occur in two well-separated clusters. Similar receptor sequences in other taxa (birds, reptiles and amphibians) have sequences, which branch off the internal edge leading to the bifurcation of mammalian GPRC5A and GPRC5D receptor sequences (Figure 1A). This finding is supported by high posterior probability and ML-bootstrap values. Given this fact, we think the correct name for these ancestral receptors is GPRC5A/D and not GPRC5A, as stated in several online databases. The sequences from *Ciona *and *Branchiostoma*, which are the most ancient sequences in our dataset, group with XRAIG4, a sequence only present in amphibians (*Xenopus*). We found XRAIG4 to be expressed in oocytes, in the eye and stomach of *Xenopus laevis*, weaker expression was detected in the liver, brain and kidney (Additional file 1, Figure S1B). The receptor sequences from lamprey form a distinct lineage.

### Evolutionary conservation of expression patterns

To date, expression patterns of GPRC5 receptors have only been published for mammals. To investigate whether GPRC5 receptor expression patterns are evolutionarily conserved, we designed primers to amplify different GPRC5 members in chicken (*Gallus gallus*) and fish (*Oryzias latipes*). Mammalian GPRC5A is expressed in highest amounts in the lung, but not in skin, and GPRC5D is expressed in skin but not in the lung tissue. Expression patterns of GPRC5 receptors were analyzed for brain and lung tissues in chicken (Figure 1B). RT-PCR analyses showed, that GPRC5A/D is expressed in high amounts in the lung of chicken and GPRC5B is expressed in high amounts in the brain. To investigate whether the GPRC5A/D sequence we found in fish (see next chapter) could be an early ancestor of GPRC5A and GPRC5D, RT-PCR analysis was used to screen various tissues from *Oryzias latipes *for mRNA expression. We found GPRC5A/D to be expressed in gills and skin, but not in other tissues investigated (Figure 1C). Highest expression of GPRC5Ba was detected in the brain of *Oryzias*, while expression of GPRC5Bb could not be detected in any of the tissues tested (for nomenclature of fish sequences see next chapter).

### GPRC5 gene duplications in fish

While GPRC5-like receptors have not been identified in fish by one study investigating presence of class C GPCRs across vertebrate and invertebrate taxa [5], GPRC5B and GPRC5C sequences were found in a survey of GPCRs in *Tetraodon nigrovirides *[17]. In the present study, we document the presence of three types of GPRC5-like receptors, confirming the presence of GPRC5B and GPRC5C in several fish species and establishing the existence of an additional sequence (GPRC5A/D).

**Figure 2:**
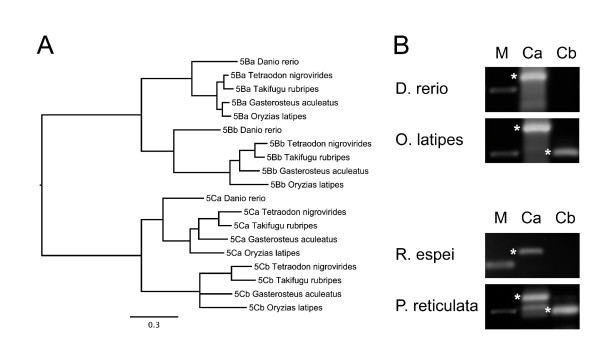
**GPRC5 receptor family in fish**. **(A) **Phylogenetic tree of all GPRC5B and GPRC5C sequences found in fish showing the separation of GPRC5Ba/GPRC5Bb, and GPRC5Ca/GPRC5Cb. Branch labels indicate posterior probabilities. Branch lengths represent phylogenetic distances. The burnin value for the Bayesian analysis was 500. **(B) **PCR with degenerate primers for amplification of GPRC5Ca and GPRC5Cb. *O. latipes *and *P. reticulata *showed a GPRC5Cb amplicon, whereas *D. rerio *and *R. espei *did not. Marker (250 bp) is indicated with M.

In addition, all bony fish in this study have two sets of GPRC5B, which share a similarity of 49-66% and shall be referred to as GPRC5Ba and GPRC5Bb receptors. We also found two classes of GPRC5C receptors (GPRC5Ca and GPRC5Cb) in four different fish species, while only one GPRC5C sequence was found in zebrafish (*Danio rerio*). This is interesting, because the zebrafish is the only fish sequenced so far that belongs to the class of Ostarioclupeomorpha (Ostariophysi), whereas the other fish belong to the Euteleostei (Acanthopterygii). The phylogenetic analysis shows that GPRC5Ca and GPRC5Cb, as well as GPRC5Ba and GPRC5Bb sequences cluster in different branches with high support values (Figure 2A, see also Figure 1).

To ascertain whether differences in the number of GPRC5 genes is specific for certain fish species, we designed degenerate primers that target conserved areas of the GPRC5C receptor sequences, but still allowed differentiation between GPRC5Ca and GPRC5Cb. Genomic DNA from *Danio rerio *and *Oryzias latipes *was used as a positive control, since GPRC5Ca and GPRC5Cb were found in the genome of *Oryzias*, while in the genome of *Danio rerio *GPRC5Ca, but not GPRC5Cb was found, although the genome project already reached maintenance stage for this organism. Analysis with isolated genomic DNA from *Rasbora espei*, a member of the Ostariophysi, and a member of Acanthopterygii (*Poecilia reticulata*) revealed that GPRC5Cb sequences are not present in *Rasbora espei*, but were detectable in *Poecilia reticulata *(Figure 2B). This finding supports to our hypothesis that two GPRC5C receptor sequences are present in Acanthopterygii and perhaps also in other Euteleostei, but not in Cyprinidae, belonging to the superorder of Ostarioclupeomorpha fish. However it must be noted, that indetectability by PCR cannot completely rule out presence of the gene. Attempts to confirm absence of GPRC5b in Cyprinidae by amplification of both genes with degenerate primers did not yield in clear results due to the frequent occurrence of unspecific amplificates.

### GPRC5 receptors in invertebrates

**Figure 3:**
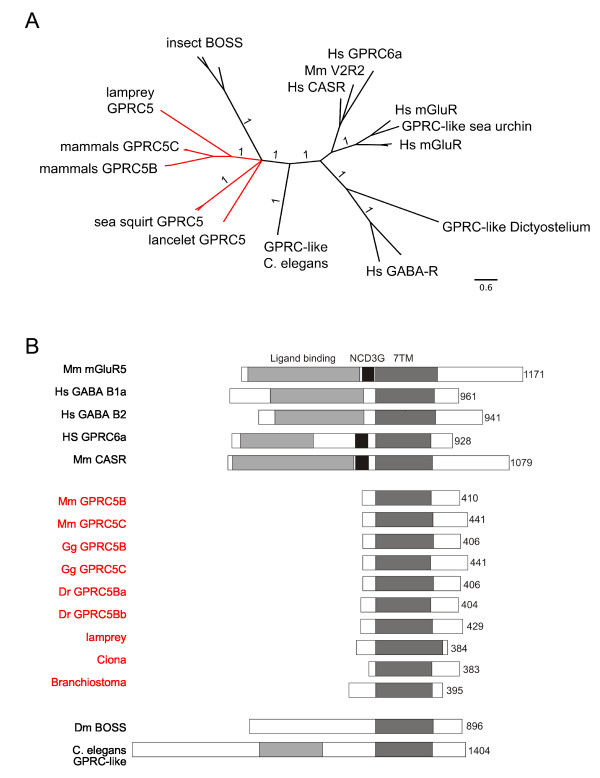
**GPRC5-like sequences in invertebrates**. (**A) **Phylogenetic tree of GPRC5-like sequences from invertebrates together with GPRC5B and GPRC5C from mammals (*Mus musculus *(Mm), *Homo sapiens *(Hs)) and calcium sensing receptors (CaSR), pheromone receptors (V2R2), metabotrope glutamate receptors (mGluR) and GABA receptors (GABA-A and GABA-B). Branch labels indicate posterior probabilities. Branch lengths represent phylogenetic distances. The burnin value for the Bayesian analysis was 500. (**B) **Domain structure of GPRC5 receptors and GPRC5-like sequences in invertebrates. Sequences are aligned at their 7TM domain areas (dark grey). The number of amino acids in each sequence is indicated on the right. GPRC5 sequences are highlighted in red in A and B.

Invertebrates have sequences that were previously classified as GPRC5-like, e.g. BOSS in *D. melanogaster *and other insects, or GPRC5B in *C. elegans *[18]. The phylogenetic analysis of the "early chordata data set" revealed, that invertebrate receptors show only low similarities to GPRC5 receptors (Figure 3A). Nevertheless, analysis of the domain structure of the encoded proteins clearly showed that unlike other class C GPCRs, all GPRC5 receptors possess short N-termini. The invertebrate receptors have long N-termini, like other class C GPCRs and unlike GPRC5 receptors in Chordata (Figure 3B ). Also in Echinodermata, which represent the closest non-chordate relatives, the closest homologue to chordate GPRC5 receptors has a long N-terminus and groups with other class C GPCRs in the alignment (Figure 3A). Furthermore, we could not identify GPRC5-like receptors in Parazoa (*Amphimedon queenslandica*) or single-celled eukaryotes such as *Monosiga brevicollis, Saccharomyces cerevisisae *or *Dictyostelium discoideum*.

The transition between N-terminus and transmembrane domain (TM) 1 of GPRC5B and GPRC5C receptors contains a stretch of amino acids which is highly conserved in all species, including urochordates but not in insect BOSS-like receptors (Additional file 2). Together our results suggest that GPRC5 receptors seem to be a chordate specific class of GPCRs.

### Intron-exon structure

**Figure 4:**
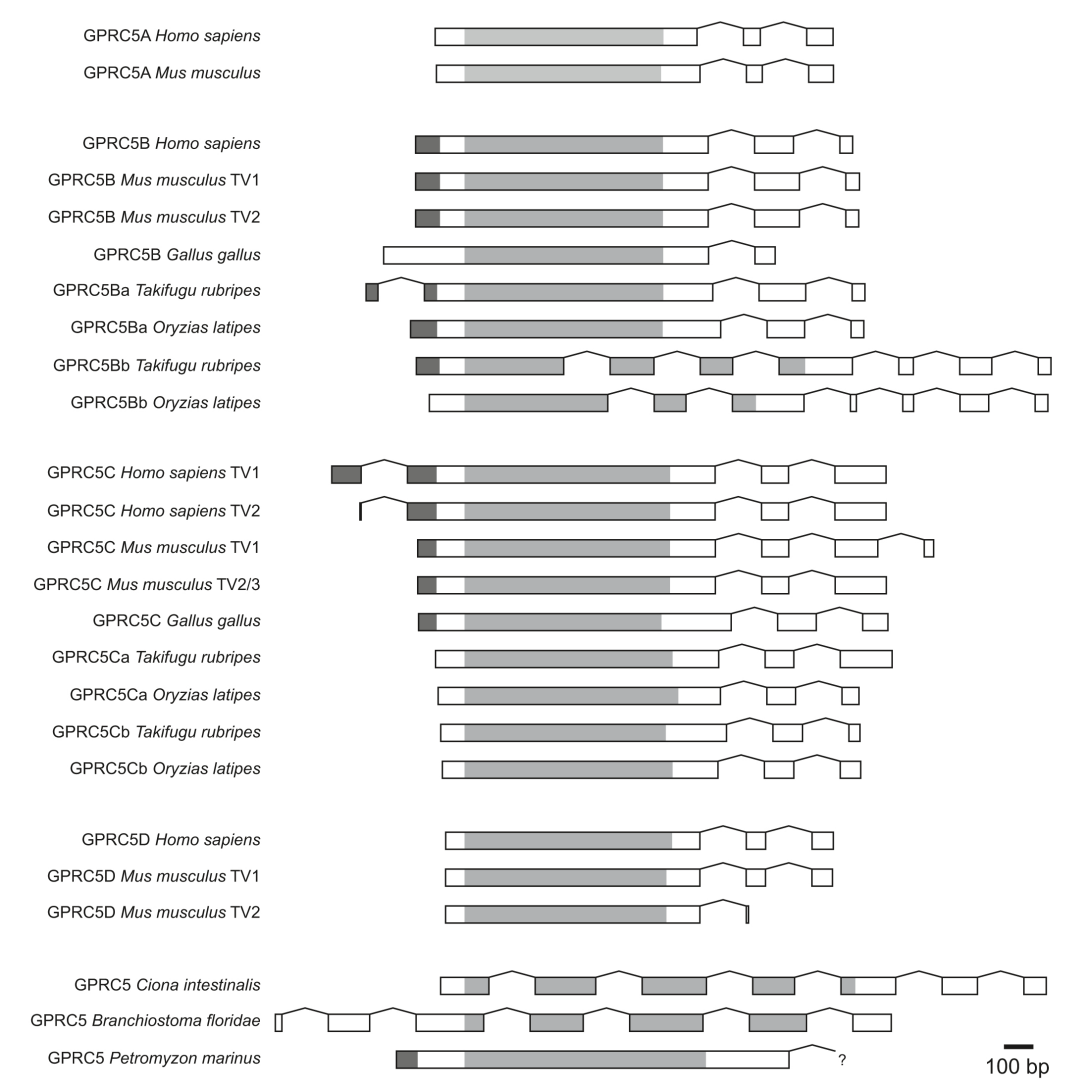
**Intron/exon structures of GPRC5 receptor sequences from different species**. The exon sizes are drawn to scale. Introns are all shown in the same size. Intron-exon structures for mouse and human are taken from the "Consensus CDS (CCDS)" project, since this database only includes protein coding regions that are consistently annotated and of high quality. "?" in *Petromyzon *GPRC5 indicates that the 3' sequence is incomplete in the database. Transmembrane domains are indicated in grey, the signal peptides (predicted with SignalP 3.0) are indicated in black.

The prevailing genomic structure of GPRC5 receptors is composed of a large exon containing the transmembrane domains, followed by two small C-terminal exons. Number and size of exons of GPRC5 genes is highly conserved among vertebrates (Figure 4). Only the duplicated GPRC5Bb fish sequences show a clearly different gene structure compared to the other GPRC5B genes. GPRC5A and GPRC5D are always located directly adjacent to each other on the respective chromosomes (Chromosome 12p3 in human, 12 in chimpanzee, 6G1 in mouse, 4q23 in rat, 5 in cow, and 27 in dog) indicating that those arose via recent gene duplication. The chromosomal regions around GPRC5 receptors are almost identical in most species (with sufficient sequence information), further indicating that the receptors arose from gene duplication events (Additional file 3). Only the organization of GPRC5 genes in *Ciona *and *Branchiostoma *differ from the organization in vertebrates.

### Sequence analysis of GPRC5 receptors

**Figure 5:**
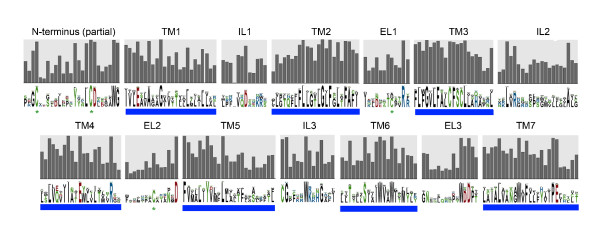
**Sequence logo plot of all GPRC5 receptor sequences**. Polar amino acids are labeled in green, basic in blue, acidic in red and hydrophobic in black. Transmembrane (TM), intracellular (IL) and extracellular (EL) loops are indicated. Transmembrane areas are additionally marked with blue lines, the degree of conservation is indicated by the bar diagram in grey.

GPRC5 receptors have short extracellular N-termini, and GPRC5B and GPRC5C protein sequences contain cleavable signal peptides (SP). GPRC5B sequences, which are also present in invertebrate species, show the highest degrees of similarity (Additional file 4, 5), despite the large phylogenetic distance between the species included in this alignment. To display patterns of sequence conservation in GPRC5 receptors we generated a sequence logo plot of all GPRC5 sequences (Figure 5), showing areas which display very high amino acid similarities. In general, the TM regions of the receptors are well conserved, especially the second, third, and seventh helices. The extracellular loops (ELs), especially EL2 and EL3 in GPRC5A and GPRC5D (Additional file 4, 5), show a lower degree of conservation than the rest of the proteins. The intracellular loops (ILs), that likely act as points of interaction with cytosolic proteins, are highly conserved.

Among the highly conserved sequence motifs is the WV(V/A)W motif in TM6, which aligns with the CWXP motif in rhodopsin and the WL motif in other class C GPCRs. A motif that is not found is the PKXY motif present in TM7 of class C GPCRs. The two cysteines in EL1 and EL2 of class C GPCRs are conserved in GPRC5B and GPRC5C, but not in GPRC5A and GPRC5D (Figure 5 and Additional file 6, Figure S6A). Moreover, we identified putative IQ motifs in GPRC5 receptors (Additional file 6, Figure S6B), which are known to be present also in other class C GPCRs.

Another motif that is conserved in all GPRC5 family members, WDD, is located at the junction of EL3 and TM7, except for GPRC5B, which has a WXD motif. At the C-terminus of GPRC5B and GPRC5C a highly conserved motif (M/V)ENKAFSMDE was identified. In IL3 we found a conserved motif composed mainly of basic amino acids at a position that is likely involved in the interaction with cytosolic proteins. A strongly conserved stretch of unknown functional significance is located approximately ten amino acids upstream of TM1 (Figure 5 and Additional file 2), where the LCD motiv at the core, followed by a conserved tryptophan, is present in all family members. Longer stretches of conserved features in the vicinity of this motiv were found in GPRC5B and GPRC5C sequences.

Nearly no conserved serines and threonines are present, which may indicate that S/T dependent phosphorylation events do not play a major role in the signaling cascades of these receptors. Furthermore, GPRC5 receptors contain neither consensus sequences for N-linked glycosylation, nor residues in their C-termini serving as putative palmitoylation sites.

### No evidence for positive selection among GPRC5 receptors

Apart from conserved sequence stretches that are found in all four GPRC5 families, some motifs are unique for specific subfamilies, e.g. in EL1 (Additional file 6, Figure S6A). Since ELs are in contact with the cell's environment, these sequence patterns might be involved in ligand binding. In this case, gene duplication could have facilitated the acquisition of novel functions (recognition of novel ligands) through the fixation of beneficial mutations, i.e., neofunctionalization. To test whether GPRC5A or GPRC5D have undergone neofunctionalization, we analyzed the evolutionary constraints that are acting on the ELs of this gene family. dN/dS ratios for the ELs of GPRC5A and GPRC5D revealed evidence for moderate to strong negative selection, no evidence for any positively selected site was found. This finding has been verified for the whole receptor sequences. Moreover, for the whole sequences it was possible to compute meaningful dN/dS rations for the individual branches connecting the outgroup and the GPRC5A as well as the GPRC5D sequences. Again no positive selection could be detected (Table 1 and Additional file 7).

**Table 1:** dN/dS values for extracellular loops (El1-El3) of GPRC5A and GPRC5D, the last column shows verification with PAML program using the whole receptor sequence

dN/dS ratio	EL1	EL2	EL3	whole receptor sequence
GPRC5A	0.129308	0.164821	0.596208	0.2146

GPRC5D	0.207865	0.144241	0.109638	0.1669

GPRC5A+D	0.269276	0.478913	0.302212	0.1842

Branch leading from outgroup to 5A group	n.n.	n.n.	n.n.	0.0708

Branch leading from outgroup to 5D group	n.n.	n.n.	n.n.	0.2333

Extracellular loops were too short to compute dN/dS rations along individual branches.

### *In vivo *expression alters in vitamin A-deficient animals

**Figure 6:**
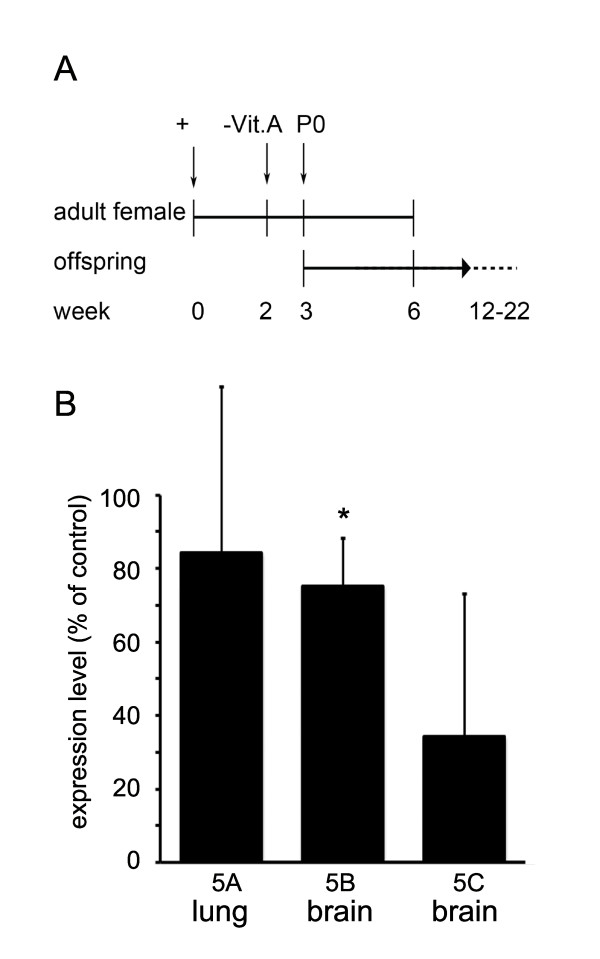
**GPRC5 genes are regulated by vitamin A**. **(A) **Schematic representation of the generation of vitamin A-deficient mice (VitA-). (**B**) Quantitative PCR analysis of GPRC5 mRNA expression levels in lung (GPRC5A) and in whole brain lysates (GPRC5B and GPRC5C). GPRC5B mRNA was significantly reduced in vitamin A deficient mice (n = 5 animals), whereas GPRC5C and GPRC5A mRNAs showed only a non-significant reduction compared to non-treated wt mice (n = 5 animals). Error bars represent SEM.

Expression of GPRC5 receptor in tumor cell lines was reported to depend on retinoic acid (RA) [10]. To test whether RA also effects GPRC5 expression under *in vivo *conditions, we produced vitamin A-deficient mice, according to the diagram shown in Figure 6A, and analyzed GPRC5 mRNA expression. When pronounced symptoms of vitamin A deficiency developed (approximately at week 22), expression of GPRC5 mRNA was quantified by real-time PCR (Figure 6B). Expression of GPRC5B in the brain was significantly decreased, while GPRC5A in the lung and GPRC5C in the brain showed non-significant regulation.

## Discussion

In this study, we identified and compared the complete repertoires of GPRC5 genes deposited to commonly accessible databases. We analyzed their phylogenetic relationships and revealed an evolutionarily conserved expression pattern, suggesting an important function of this receptor family in chordate evolution. A possible evolutionary scenario for the vertebrate GPRC5 gene family was derived from the results of these analyses.

### GPRC5 receptor families in different species

Several families of GPRC5 genes were identified in all major classes of chordate species, as well as in the sea lamprey, an example of a jawless vertebrate. We did not detect pseudogenes in any of the analyzed animals. In simple chordate species, such as amphioxus and sea squirts, we found one GPRC5-like receptor, no GPRC5C-like sequences were identified in more basal Deuterostomia species, Protostomia or more primitive Eumeatazoa. Given these facts, we hypothesize that the receptors are present exclusively in chordate species.

Bayesian and RAxML analyses of the identified sequences (Figure 1, Additional file 1) showed that all receptor sequences form four distinct families, GPRC5A-D, that have been identified previously in mammals [5]. Not all sequences included in the phylogenetic trees were available in full length in the databases, meaning that small modifications of the tree might be possible once the entire sequences are available. Since we included only receptors with high sequence coverage (> 95%), major changes are unlikely to occur.

The sequences from *Ciona*, amphioxus and *Petromyzon *constitute a separate branch with similarities to GPRC5C receptors from vertebrates. The *Xenopus *RAIG4 sequence clusters with the sequences from species from early diverging lineages. The receptor was previously described as XRAIG4a, together with a close homolog called XRAIG4b in *Xenopus laevis*. XRAIG4a and XRAIG4b were classified as pseudoalleles due to gene duplication in the *Xenopus laevis *lineage [16], but could also represent splice variants or even sequencing errors (Additional file 8). Since we did not find XRAIG4-like sequences in other species, the receptors could be a divergent ortholog, grouping with the other long branches of the non-vertebrate chordates. Since *Xenopus*-specific duplications were also found in other protein families [19], XRAIG4 could originate from an amphibian (or *Xenopus*) specific duplication of GPCR5C. Alternatively, it could be an "Ohnolog gone missing" [20] from all other vertebrate lineages (which express only 3 receptor types after 2 rounds of genome duplication). Both scenarios cannot be unambiguously distinguished at the moment.

### GPRC5 family in teleost fish

Although GPRC5 receptor genes were not identified in fish in a previous study [5], we found that the number of putatively functional GPRC5 genes in teleost fish (ray-finned fish) is actually larger than in tetrapods due to the presence of additional receptor sequences that share similarities with GPRC5B and GPRC5C, named GPRC5Bb and GPRC5Cb in this study (Figure 2). These additional receptor genes may result from the 3R teleost-specific genome duplication events, which occurred in an ancestor of the teleost lineage around 300-350 million years ago [21]. In addition we found sequences denoted as GPRC5A/D, which are clearly located on the branch leading to the bifurcation of mammalian GPRC5A and GPRC5D receptor families in the Bayesian and the ML tree (supported by high posterior probabilities and ML bootstrap values). Interestingly, we found GPRC5A/D genes only in Acanthopterygii, and not in Ostariophysi, although sequence information is limited due to the fact that *Danio rerio *is the only species sequenced from the latter class.

While GPRC5Bb receptors were found in all teleost fish species investigated, additional GPRC5Cb sequences were only detected in the genomes of *Oryzia latipes, Gasterosteus aculeatus, Takifugu rubripes *and *Tetraodon nigrovirides*, all belonging to the superorder Acanthopterygii. Genomic analysis using degenerate primers, designed to amplify GPRC5B and GPRC5C receptor sequences from not yet fully sequenced fish, supports our finding that Acanthopterygii have two GPRC5C sequences, while different members of the Cyprinidae family (belonging to superorder Ostariophysi) may have only one. GPRC5Cb genes may have been lost during the speciation process in some teleost species (Cyprinidae), while being retained in others (belonging to the superorder Acanthopterygii).

It is interesting to note that also the number of orthologues to the mammalian glutamate receptors is much lower in *Danio rerio *than in *Takifugu rubripes *[5]. It is noteworthy that tetraploidization in teleost fish did not increase the number of other class C GPCRs such as TAS1R, GABA, GPCR6 and CASR. So far the only known gene duplication in this group led to duplication of glutamate receptor 1 in fugu and glutamate receptor 2 in fugu and zebrafish. Thus, the proposed tetraploidization events in teleosts did not have a major effect on the repertoire of the class C family of GPCRs, except for GPRC5 receptors.

### Origin of GPRC5 receptors

**Figure 7:**
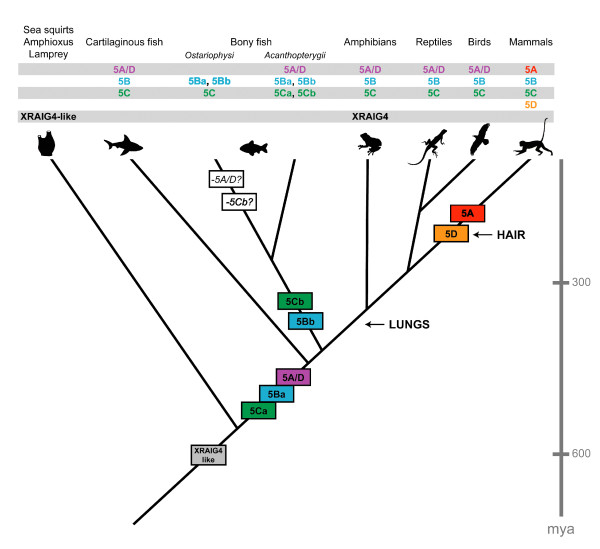
**Evolution of GPRC5 receptors**. Scale bar in million years (mya). Basal taxa (sea squirts, amphioxus and lamprey) have receptor sequences similar to XRAIG4, which is present in frogs, but not in all other vertebrate species. All vertebrates have genes for GPRC5B, GPRC5C and GPRC5A/D, only some bony fish (Ostariophysi) seem to have lost the 5A/D receptor sequence. GPRC5B is duplicated in all bony fish, GPRC5C is duplicated in Acanthopterygii fish. GPRC5A retains at least one ancestral (or plesiomorphic) tetrapod expression domain, in lungs, while GPRC5D may have recruited an ancestral vertebrate skin expression domain into derived, mammalian structures (hair).

It has been proposed by Susumu Ohno [22] that series of large-scale whole-genome duplications occurred on the vertebrate stem lineage providing the genomic basis for the evolution of vertebrate complexity. Comparison of sequences from sea squirt, lamprey, elephant shark and several bony fish revealed that two rounds of duplication have probably taken place and constrained the timing of the whole-genome events to after the divergence of vertebrates from tunicates and lancelets, but before the split between cartilaginous fishes and bony vertebrates [23]. Within the resolution of our analysis, we found evidence that the genes that arose from duplication of the evolutionarily early GPRC5C-like genes are retained in vertebrate genomes, followed by a loss of one gene early in the vertebrate lineage (summarized in Figure 7). We found three GPRC5-like receptors in all vertebrates, indicating that the genes indeed stem from the early duplication events at the vertebrate stem. The presence of two genes in lamprey is consistent with the hypothesis that lamprey radiated after the first round of these whole-genome duplications. Regarding the position of both lamprey sequences together on their own branch of the phylogenetic tree, an alternative scenario could be that if lamprey shared one of the ancient duplications, the ancient GPRC5 paralog may have been lost, followed by an independent genome duplication in this lineage [24]. The same event could have led to two lamprey genes, even if lamprey did not undergo the first round of whole genome duplication in the vertebrate lineage. The fact that the *Ciona *genomes contain only one GPRC5-like sequence implies that the GPRC5 genes originate from the rounds of duplication after the divergence of the urochordate lineage.

The fact that three of the GPRC5 genes are retained in vertebrates is remarkable, as sequencing of the human and other vertebrate genomes has shown that most gene duplicates from whole-genome duplication events were lost rapidly, since gene numbers in vertebrates is comparable to that of invertebrates [25]. What is typically retained in modern genomes is a disproportionate number of genes involved in developmental processes, which are significantly enriched for functions associated with signal transduction, transcriptional regulation and neuronal activities. Genes implicated in signal transduction are more than twice as likely to be retained in two or more copies compared to the overall retention rate [23].

### Evolution of gene function

Duplicated genes often encode protein products that, although not essential for viability of the organism, are important for the adaptation of the species to specific ecological niches [26]. Although not much is known about the functions of the receptors to confirm their role in vertebrate development, the expression patterns seem to be evolutionarily conserved. GPRC5B shows a neuronal expression pattern in mammals [10,11], which we could also find in birds and fish. All species for which GPRC5B-like sequences were detected have a complex central nervous system.

GPRC5A/D is expressed in the lung of different tetrapods and in the gills and skin of teleosts. The mammalian GPRC5A/D paralogs arose from gene duplication when or shortly after mammals diverged, but before the radiation of the eutherian (placental) orders of mammals. GPRC5A is known to be expressed in mammalian lungs [27,28], and although it has been shown in knock-out mice that GPRC5A is not necessary for normal lung development (for the parameters investigated) [8,28], loss of GPRC5A expression increases the incidence of lung tumors [28]. On the other hand, reduction of GPRC5A expression was found to be associated with reduced growth in different cell types [29,30].

GPRC5D is expressed in differentiating cells that produce hard keratin and have been suggested to be involved in the regulation of keratin synthesis in hard epithelial tissues [12]. All mammals have hairy skin or have evolved from ancestors that had hair, which was necessary for all mammals to save energy while maintaining their body temperature and for protection from UV light. Although also birds and reptiles have hard appendages (claws, feathers, scales), the molecular composition of these structures is different from mammals. A GPRC5D sequence is also present in dolphin (*T. truncatus*), which, as all hairless mammals that live in the sea, is thought to be a descendent of the Mesonyx, a hairy, dog-like animal that developed back to a sea-living creature during evolution. The dolphin GPRC5D sequence shows a couple of unique changes in the amino acid sequence compared to the mammalian GPRC5D consensus sequence (Additional file 9) which might indicate functional changes of the protein.

Several models have been proposed to explain why genes might survive after whole genome duplication [31]. Sequence analysis of the subtype specific sequences of EL1 revealed similarities between GPRC5A and GPRC5D (conserved proline residue at the C-terminal end of the loop) and marked differences reflected by the loss of charged amino acids in GPRC5D (present in GPRC5A/D and GPRC5A). This could in principle lead to differences in function of the duplicated receptors, such as, e.g., different activating ligands. According to the neofunctionalization model, one would expect to detect signs of positive selection in the loop sequences based on the analysis of dN/dS ratios, which was not the case. Since dN/dS ratios are not always reliable indicators of positive selection in the case of highly similar sequences, final interpretation of the results is difficult. Another concern is that episodic positive selection could have operated only around a certain time window, whereas over the majority of the evolutionary time the genes could have been dominated by selective constraints.

According to the subfunctionalization model, duplicated gene copies specialize to perform complementary functions. The fact that mammalian GPRC5A and GPRC5D show restricted expression patterns in certain tissues (lung/skin) would be consistent with paralogs that do not have different functions, but partitioned expression patterns. This scenario is supported by the fact that the teleost GPRC5A/D receptor is expressed in skin and gills. Since we do not know whether GPRC5A/D is expressed in parts of the gill of endodermal origin, we cannot draw conclusions about putative similarities to the expression of GPRC5A in the mammalian lung, also being of endodermal origin, although expression of the receptors in endodermal structures might be evolutionarily conserved. Closer histological investigation of the expression of GPRC5A/D in gills and analysis of the expression of GPRC5A/D receptors in the skin of non-mammalian tetrapods could be performed in the future to strengthen the subfunctionalization model.

In the balanced gene drive model, duplicates are retained because the gene dosage change results in increased fitness. Since GPRC5C seems to be relatively ubiquitously expressed, coexpression of GPRC5B, GPRC5A, and GPRC5D may specifically increase the gene dosage in some tissues. To further investigate this model, it would be necessary to investigate the expression strength, the subcellular localization and the physiology of the different proteins.

### Function of GPRC5 receptors

GPRC5 receptors have also been named RAIGs, since expression of the respective mRNAs is induced in tumor cell lines by stimulation with RA. We showed here that expression of GPRC5B is significantly down-regulated in the brain of mice fed a RA-deficient diet. Expression of GPRC5 receptors is therefore regulated, directly or due to a secondary effect by RA *in vivo*. We did not detect a significant regulation of GPRC5A in the lung, and GPRC5C in the brain of these animals, although expression levels seem to be reduced. RA, a physiologically active form of vitamin A, regulates gene expression by binding to a complex of retinoic acid receptors (RARs) and retinoid X receptors (RXRs). Expression of RARα was found to be synchronized with that of GPRC5B, indicating the involvement of GPRC5B in RA-dependent morphogenesis/angiogenesis [13]. Induction of receptor expression was found to be mediated by a functional RAR/RXR binding site identified in the proximal 5' upstream region of the GPRC5A gene [14]. Since RA plays crucial roles in many physiological events, such as embryonic development, reproduction, metabolism and homeostasis [15], RAIGs are considered essential to RA-dependent physiological events.

Because the action of RA during development was regarded as being restricted to chordates for a long time, it had been proposed that development of RA-binding nuclear hormone receptors and the RA-synthesizing and RA-degrading enzymes was an important step for the origin of the chordate body plan [31]. Interestingly, the larvacean urochordate *Oikopleura dioica*, which maintains a chordate body plan throughout life, lacks genes for RA synthesis, degradation, and reception [32]. Although we found GPRC5 receptors in two urochordate species (sea squirts), we did not find GPRC5-like receptors in the genome of *Oikopleura*, also belonging to the class of Urochordata. This suggests that the RA-machinery was lost during larvacean evolution and predicts that *Oikopleura *development has become independent of RA-signaling.

## Conclusions

The vertebrate GPRC5 receptors GPRC5C, GPRC5B and GPRC5A/D evolved from a receptor present in basal chordate species, which has no clear homolog in invertebrates. One of these sequences, GPRC5A/D, has duplicated into GPRC5A and GPRC5D receptors in mammals. The receptors show conserved expression patterns in lung (GPRC5A), hairy skin (GPRC5D) and the nervous system (GPRC5B). Due to their regulation by the RA signaling cascade, their high degree of conservation, and their specific expression patterns, this receptor class forms an interesting target for future studies.

## Methods

### Phylogenetic analysis

In this study, three data sets have been analyzed, namely the "*main GPRC5 data set" *with 143 sequences (Figure 1, Additional files 1, 10), the "*B-B2-C-C2 data set" *consisting of 19 receptor sequences of taxa that were found to have an additional copy of the GPRC5B and/or the GPRC5C receptors (Figure 2). This data set is a true subset of the main GPRC5 data set. The "*invertebrate data set*" consists of 26 receptor sequences, including sequences from invertebrates that have homologies to GPRC5 receptors (Additional file 11). The MAFFT alignments of the three data sets used for phylogenetic reconstructions had the following main characteristics: "main data set": 143 sequences, 809 amino acids, B-B2-C-C2 data set: 19 sequences, 667 amino acids, "invertebrate data set": 26 sequences, 2309 amino acids. All three data sets of amino acid sequences have been aligned independently using MAFFT version 6 [33,34] with the E-INS-I algorithm using the MAFFT-plugin in the Geneious software. An appropriate amino acid substitution model was determined using ProtTest version 2.4 [35]. MrBayes version 3.1.2 [36] and RAxML versions 7.0.4 and 7.2.7 [37] were employed to estimate phylogenetic trees. Bayesian analyses of all three data sets were conducted by computing 10 × 10^6 ^Markov Chain Monte Carlo generations in two parallel runs, each with four chains. Trees were sampled every 100 generations. Convergence was determined for both runs. For the "main data set", the most likely tree was determined with RAxML by conducting 30 ML searches on 30 randomized stepwise addition parsimony trees. Furthermore, a total of 4,000 normal bootstrap replicates have been computed with RAxML. They were used (i) to compute a majority rule consensus tree and (ii) to map support values on the best tree. All phylogenetic analyses were performed with protein sequences, to avoid errors due to multiple substitutions and homoplasic characters present in nucleotide sequences.

The global dN/dS ratios for the extracellular loops of GPRC5A and GPRC5D receptor coding sequences were determined by using the HyPhy package http://www.datamonkey.org. These values have been verified with the codeml program from the PAML package according to methods described in [38]. To make inferences about selective pressure (positive and negative selection) on individual codons (sites) within the coding sequences, the Single Likelihood Ancestor Counting (SLAC) package was used (http://www.datamonkey.org).

### Search for novel GPRC5 sequences

Blastn, blastp and tblastn searches were performed (on http://www.ensembl.org) against genomes of various species using as query all known GPRC5 sequences available at this time. Subsequently, we aligned all GPRC5A-D sequences and used conserved sequence motifs detected to run further blast searches. We examined whole genome sequence assemblies obtained from the ENSEMBL website including eight primates, eight rodents, 16 other mammals, two birds, one reptile, five fish and one amphibian species. We also investigated genomes from: lamprey (*Petromyzon marinus*, data was produced by the Genome Center at Washington University School of Medicine in St. Louis and can be obtained from http://genome.ucsc.edu/), elephant shark (*Callorhinchus milii*, http://esharkgenome.imcb.a-star.edu.sg, Assembly: Eshark 1.4X [39], Amphioxus (*Branchiostoma floridae*, http://genome.jgi-psf.org, Assembly v1.0 [23], *Oikopleura dioica *http://www.genoscope.cns.fr/externe/GenomeBrowser/Oikopleura/, Medaka (*Oryzias latipes*, http://genome.ucsc.edu, Assembly: Oct. 2005 NIG/UT MEDAKA1/oryLat2, produced by the National Institute of Genetics (NIG) and the University of Tokyo), *Xenopus tropicalis *(http://genome.jgi-psf.org, Assembly v4.1), sea urchin (*Strongylocentrotus purpuratus *(http://www.spbase.org/SpBase/, v2.5), acorn worm *(Saccoglossus kowaleyskii*, http://www.hgsc.bcm.tmc.edu/project-species-o-Acorn%20worm.hgsc), draft assembly Skow_1.0, sea anemone (*Nematostella vectensis*, http://genome.jgi-psf.org), sponges (*Amphimedon queenslandica*, http://www.metazome.net/amphimedon, 9× assembly), choanoflagellates (*Monosiga brevicollis*, http://genome.jgi-psf.org/, v1.0), *Dictyostelium discoideum *(http://www.ncbi.nlm.nih.gov/projects/genome/guide/dicty/, 2.1); accession numbers of the sequences are listed in Additional file 12.

For intron-exon analysis of mouse and human sequences, the consensus CDS (CCDS) project was used (http://www.ncbi.nlm.nih.gov/CCDS/). Signal peptides were predicted with SignalP 3.0 (http://www.cbs.dtu.dk/services/SignalP/), transmembrane domains were predicted with TMHMM v2.0 http://www.cbs.dtu.dk/services/TMHMM/).

### Animals used for tissue preparation and PCR Analysis

DNA was prepared from zebrafish (*Danio rerio*), medaka (*Oryzias latipes*), rasbora (*Rasbora espei*) and guppy (*Poecilia reticulata*). RNA was prepared from frog (*Xenopus laevis*), chicken (*Gallus gallus*), mouse (*Mus musculus*) and medaka (*Oryzias latipes*). mRNA was prepared using RNeasy kits (Qiagen), including DNaseI digestion. Genomic DNA from fish was isolated with the QIAamp DNA kit (Qiagen). The primers used were the following (numbers indicate amplicon size): Fish: 5Bb-Fish-410-fw: MGAAGCGCAGCGGYRTSG; 5Bb-Fish-410-rev: CTCCCTCAGCACGGTSAG; 5Ba-Fish-409-fw: GACCTGGAGKCRRTVTGGGG; 5Ba-Fish-409-rev: CCCTGNACNAGCAARCWSGARAARC; 5Ca-Fish-305-fw: CTGCTGGCCRGCSTKCCCTTC; 5Cb-Fish-305-rev: GTGTTGATGAYYACYTCCACC; 5Cb-Fish-240-fw: CTTYACSCTSGGACTSTTYGG; 5Cb-Fish-240-rev: GCCAYTCNGTRTTGATGATNACCTC; 5A/D Oryzias-fw: GCCTTCTCCTGCCTGCTGGC; 5A/D Oryzias-rev: AGAGCAGGAGCGGCACAGGA; Actb Oryzias-fw: CTGCGTCTGGACTTGGCCGG; Actb Oryzias-rev: GGCCTCTGGGCAACGGAACC; chicken: 5A/D-chic-256-fw: GAAGAGACACGGAGCACACA; 5A/D-chic-256-rev: GAATGTGGGTTGGCAGAAGT; 5B-chic-254-fw: AATAGTTGTGGAGGCGGTTG; 5B-chic-254-rev: GCAAGCATGAGAAGCACAAA; actb-chic-235-fw: ATGAAGCCCAGAGCAAAAGA; actb-chic-235-rev: ACATACATGGCTGGGGTGTT. Frog primers: 5A/D-xenopus-231-fw: GCTTTAGTAGCCAACGGCTG;5A/D-xenopus-231-rev: GGCAATGGTAGCATCGTTCT; 5B-xenopus-234-fw: AGGTCCACTGCACCATTCTC; 5B-xenopus-234-rev:GGTACACGTTGCTTCGGAAT; XRAIG4-fw: AGTCGGGATGTCCCGTCC; XRAIG4-rev: GATAGCTGATGGAAGG; ODC-xenopus-386-fw: GTCAATGATGGAGTGTATGGATC;ODC-xenopus-386-rev: TCCATTCCGCTCTCCTGAGCAC.

### cDNA preparation and quantitative PCR analysis

RNA was isolated with RNeasy Mini Kits (Qiagen) from freshly prepared tissues. cDNA was synthesized with the iScript cDNA synthesis kit (Biorad). qPCR was performed with iQ™ SYBR™ Supermix (Biorad) and standard parameters (5 min 95°C, then 40 cycles with 1 minute 95°C, 1 minute 60°C and 1 minute 72°C. At the end a final 72°C step was performed for 10 minutes). All PCR products were sequenced to verify target amplification. Sequencing was carried out by the Department of Biochemistry, Ruhr-University Bochum. Experiments were carried out in triplets, with 5 animals each. mRNA expression levels were calculated according to the Pfaffl method [40]. Detailed information regarding qPCR including sample preparation/handling are listed according to MIQE (Minimum Information for Publication of Quantitative Real-Time PCR Experiments) (Additional file 12).

Primers used for qPCR: GPRC5A-fw: TTGGTTTTCTGTGGATCCTTCT; GPRC5A-rw: GGCAAGATATAAAATGCCAG; GPRB5B-fw: TTCCGGAGATCCACTACACC; GPRC5B-rw: TCTTTGCTCCAAGCTTCCAT; GPRC5C-fw: CTTCCATTTGTGCAGGACACTA; GPRC5C-rw: GAAAGGACGTGAGCTACCAG; Actb-fw: GATCATTGCTCCTCCTGAGC; Actb-rw: GAAAGGGTGTAAAACGCAGC.

### Vitamin A-deficient mice

Vitamin A-deficient mice were produced as described previously [41]. Briefly, starting 2 weeks *post coitum *pregnant female C57/BL6 mice were fed a diet containing no sources or precursors of vitamin A (VAD diet, C1016). A second mouse group received a control diet (C1000, both from Altromin, Germany). At three weeks of age pups were separated from their mother and fed the respective diet. First signs of vitamin A deficiency were observed at 18 weeks of age, pronounced symptoms at week 22. All animal experiments were in accordance with the German Guidelines for Animal Care and Use, approved by an ethics committee and the LANUV NRW.

## Abbreviations

RA: retinoic acid; GPCR: G-protein coupled receptor; TM: transmembrane domain; EL: extracellular loop; IL: intracellular loop; RT: reverse transcription; RAIG: retinoic acid inducible gene.

## Authors' contributions

EMN and SK conceived the study, designed experiments and wrote the manuscript. SK and TP carried out the molecular biological experiments, SK, TP and EMN did sequence analyses/alignment and database mining. CM and FL carried out phylogenetic analyses and helped discussing the results. HH supervised part of the work. All authors read and approved the final manuscript.

## Supplementary Material

Additional file 1 **Phylogenetic analysis**. **(A) **Phylogenetic tree obtained by RAxML. (**B**) Screening of XRAIG4 expression in different tissues from *Xenopus laevis *by RT-PCR. Click here for file

Additional file 2 **Partial GPRC5 alignments**. Alignment showing a sequence between the end of the N-terminus and the transition into transmembrane region 1 from different GPRC5 receptors. Click here for file

Additional file 3 **Analysis of the neighboring genomic regions surrounding of GPRC5 receptors**. Genes in the vicinity of GPRC5 receptors from different species. Click here for file

Additional file 4 **Sequence logos**. Sequence logos of all GPRC5 receptor sequences found in mammals. Click here for file

Additional file 5 **Hydropathy plots**. Hydropathy plots and degree of conservation of GPRC5 consensus sequences from different species. Click here for file

Additional file 6 **Sequence analysis of GPRC5 receptors**. (**A) **Alignment of the first extracellular loops from different classes of GPRC5 receptors. (**B) **Putative IQ motifs in GPRC5 receptors. Click here for file

Additional file 7 **Negative selection sites in GPRC5 receptors**. GPRC5A and GPRC5D sequences from *Mus musculus *with marked sites where negative selection was predicted. Click here for file

Additional file 8 **mRNA sequences of *Xenopus laevis *sequences**. Alignment of available mRNA sequences from XRAIG4 and GPRC5 receptors from *Xenopus laevis*. Click here for file

Additional file 9 **GPRC5D from dolphin**. Alignment of GPRC5D from *Tursiops truncatus *with the consensus of all mammalian GPRC5D sequences. Click here for file

Additional file 10 **GPRC5alignment.nex**. Alignment of all sequences included in the study. Click here for file

Additional file 11 **alignment **(Figure 3)**.nex**. Alignment used to generate Figure 3A. Click here for file

Additional file 12 **Additional Materials**. Figure legends of additional files, list of accession numbers of sequences included in this study, qPCR information. Click here for file
