# Supplementary figures and images for: Molecular evolution of a chordate specific family of G protein-coupled receptors
Source: BMC Evol Biol. 2011 Aug 9;11:234. doi: 10.1186/1471-2148-11-234 (PMC3238225; doi:10.1186/1471-2148-11-234)

**A**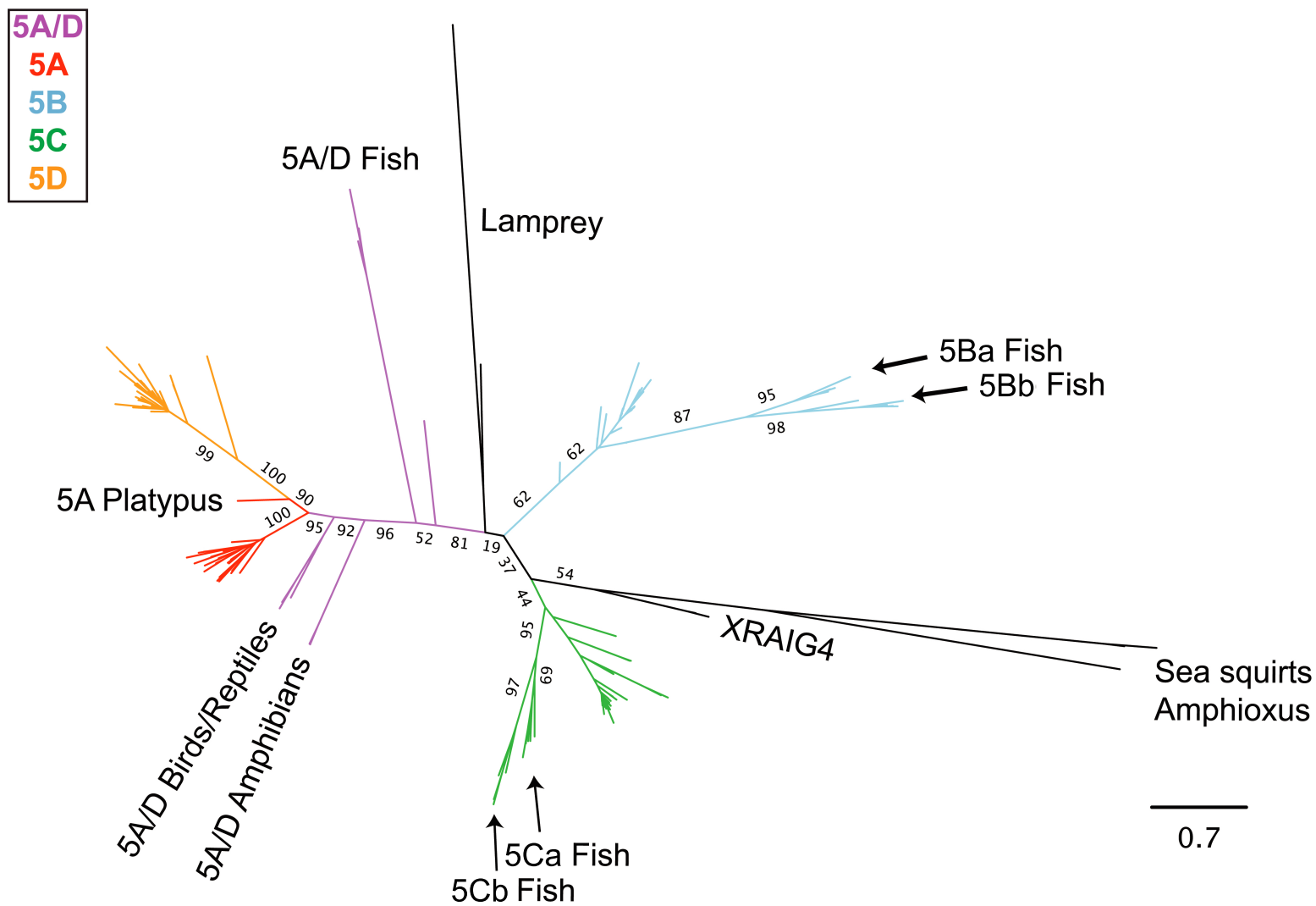**B**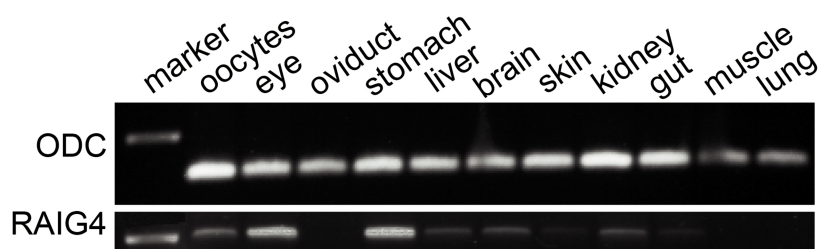

Supplement: Additional file 1 — Phylogenetic analysis. (A) Phylogenetic tree obtained by RAxML. (B) Screening of XRAIG4 expression in different tissues from Xenopus laevis by RT-PCR. [file 1471-2148-11-234-S1.pdf]

# Additional file 4

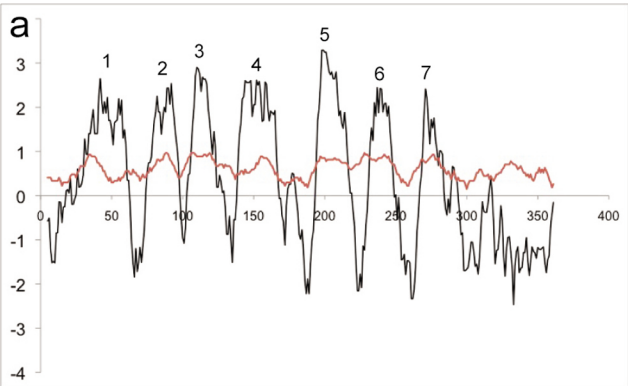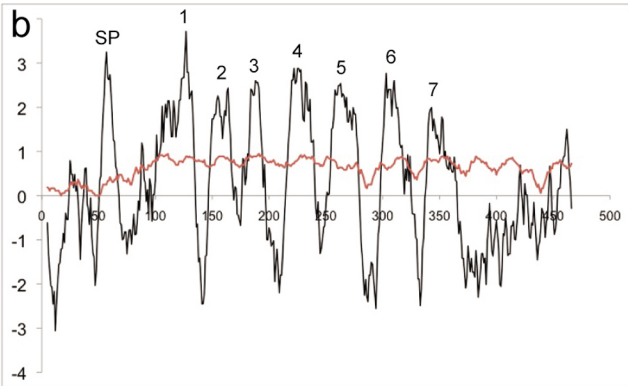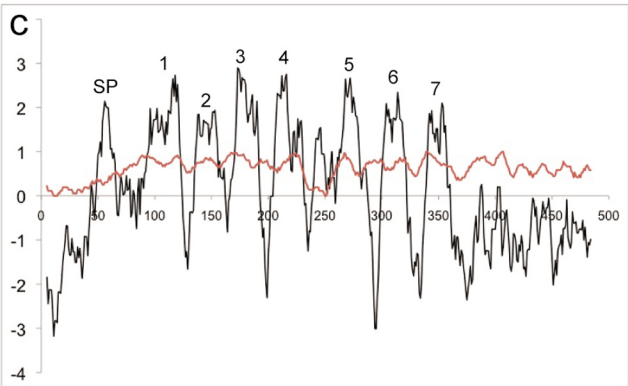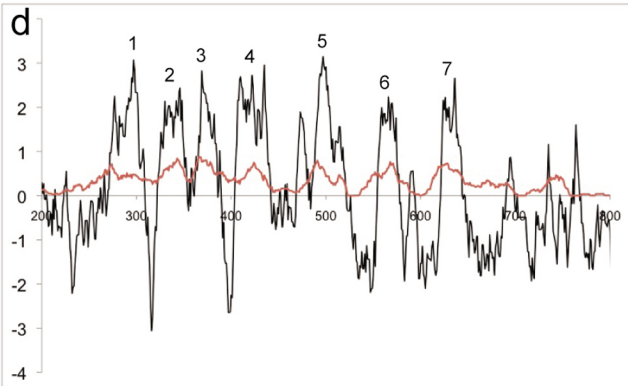

Supplement: Additional file 4 — Sequence logos. Sequence logos of all GPRC5 receptor sequences found in mammals. [file 1471-2148-11-234-S4.pdf]

GPRC5A

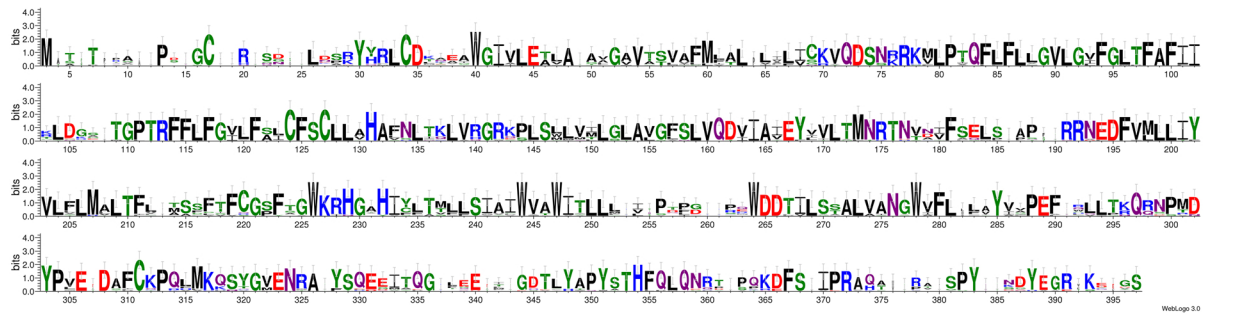

GPRC5B

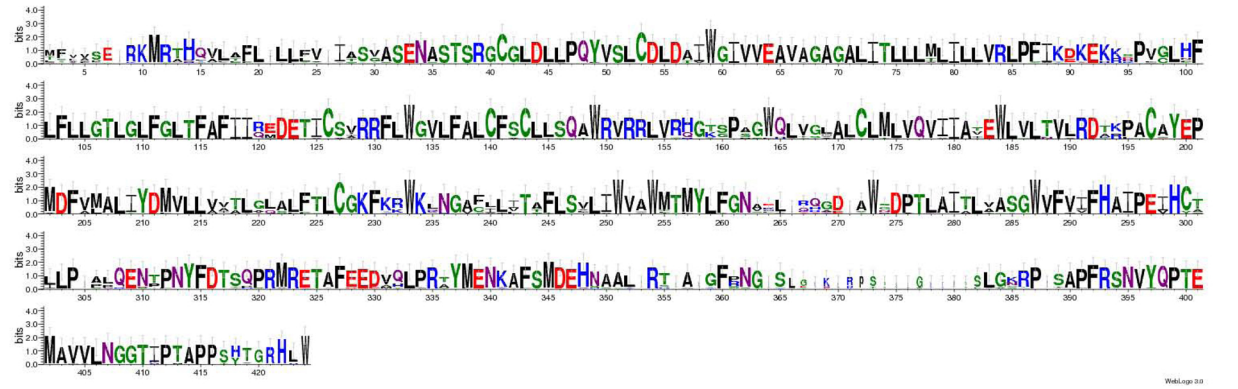

GPRC5C

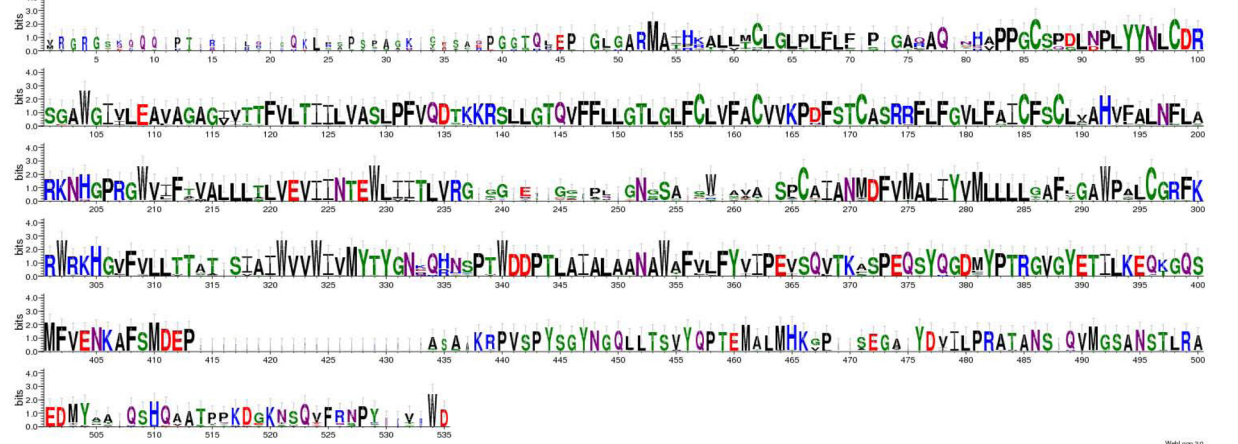

GPRC5D

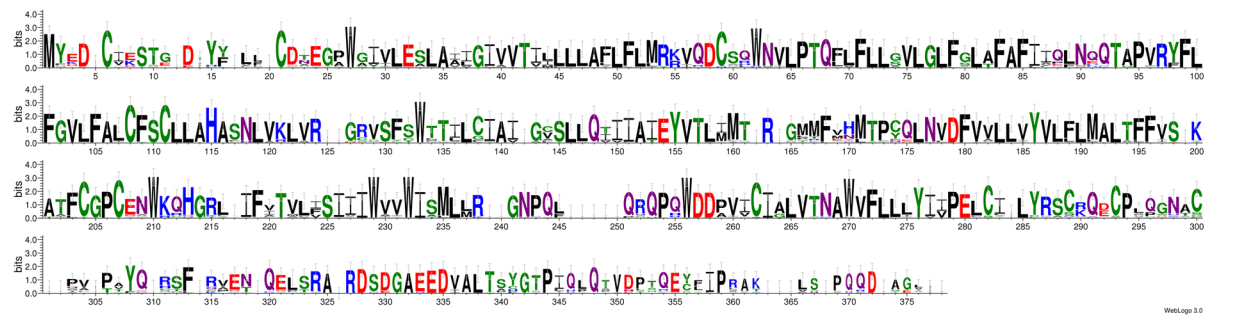

Supplement: Additional file 5 — Hydropathy plots. Hydropathy plots and degree of conservation of GPRC5 consensus sequences from different species. [file 1471-2148-11-234-S5.pdf]
