# Supplementary material for: Molecular evolution of a chordate specific family of G protein-coupled receptors
Source: BMC Evol Biol. 2011 Aug 9;11:234. doi: 10.1186/1471-2148-11-234 (PMC3238225; doi:10.1186/1471-2148-11-234)
Supplement: Additional file 2 — Partial GPRC5 alignments. Alignment showing a sequence between the end of the N-terminus and the transition into transmembrane region 1 from different GPRC5 receptors. [file 1471-2148-11-234-S2.pdf]

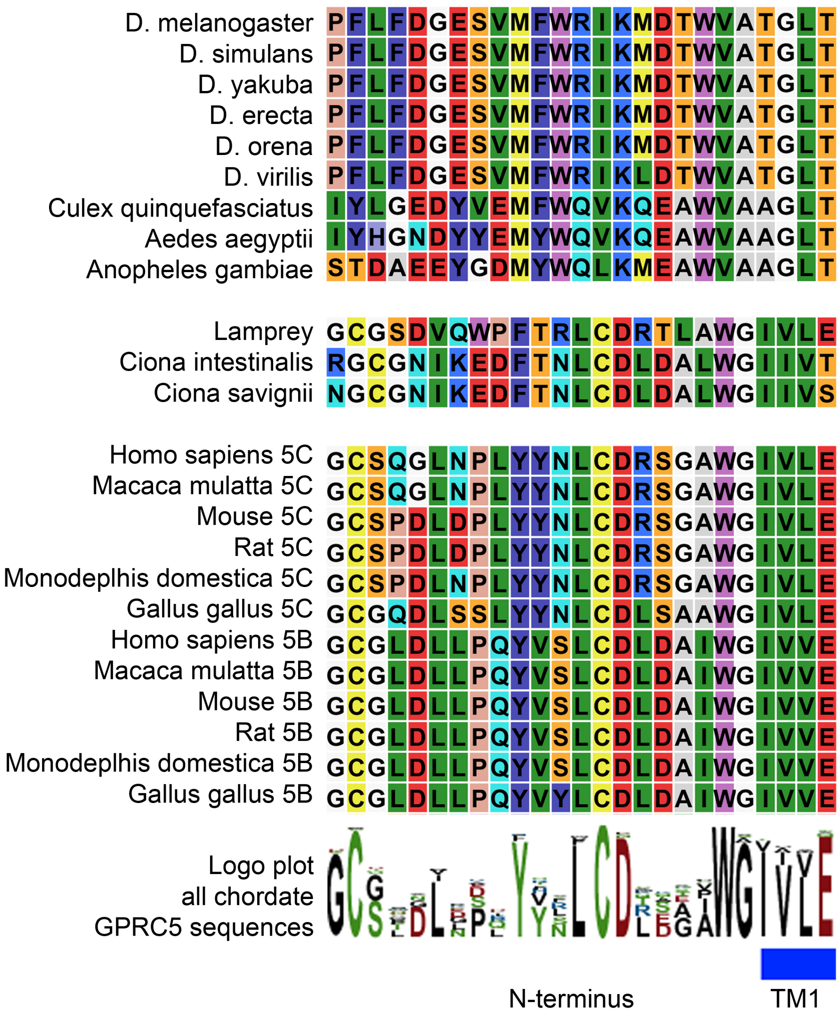
D. melanogaster PFLFDGESVMFWRIKMDTWVATGLT
D. simulans PFLFDGESVMFWRIKMDTWVATGLT
D. yakuba PFLFDGESVMFWRIKMDTWVATGLT
D. erecta PFLFDGESVMFWRIKMDTWVATGLT
D. orena PFLFDGESVMFWRIKMDTWVATGLT
D. virilis PFLFDGESVMFWRIKLDTWVATGLT
Culex quinquefasciatus IYLGEDYVEMFWQVKQEAWVAAGLT
Aedes aegyptii IYHGNDYYEMYWQVKQEAWVAAGLT
Anopheles gambiae STDAEEYGDMYWQLKMEAWVAAGLT
Lamprey GCGSDVQWPFTRLCDRTLAWGIVLE
Ciona intestinalis RGCGNIKEDFTNLCDLDALWGIIVT
Ciona savignii NGCGNIKEDFTNLCDLDALWGIIVS
Homo sapiens 5C GCSQGLNPLYYNLCDRSGAWGIVLE
Macaca mulatta 5C GCSQGLNPLYYNLCDRSGAWGIVLE
Mouse 5C GCSPDLDPLYYNLCDRSGAWGIVLE
Rat 5C GCSPDLDPLYYNLCDRSGAWGIVLE
Monodeplhis domestica 5C GCSPDLNPLYYNLCDRSGAWGIVLE
Gallus gallus 5C GCGQDLSSLYYNLCDLSAAWGIVLE
Homo sapiens 5B GCGLDLLPQYVSLCDLDAIWGIVVE
Macaca mulatta 5B GCGLDLLPQYVSLCDLDAIWGIVVE
Mouse 5B GCGLDLLPQYVSLCDLDAIWGIVVE
Rat 5B GCGLDLLPQYVSLCDLDAIWGIVVE
Monodeplhis domestica 5B GCGLDLLPQYVSLCDLDAIWGIVVE
Gallus gallus 5B GCGLDLLPQYVYLCDLDAIWGIVVE
Logo plot
all chordate
GPRC5 sequences
N-terminus
TM1
