# Supplementary material for: Molecular evolution of a chordate specific family of G protein-coupled receptors
Source: BMC Evol Biol. 2011 Aug 9;11:234. doi: 10.1186/1471-2148-11-234 (PMC3238225; doi:10.1186/1471-2148-11-234)
Supplement: Additional file 3 — Analysis of the neighboring genomic regions surrounding of GPRC5 receptors. Genes in the vicinity of GPRC5 receptors from different species. [file 1471-2148-11-234-S3.pdf]

GPRC5C

Homo sapiens	**Dna I2**	**KIF19**	**BTB(POZ) domain containing 17**	**GPR142**		**GPRC5C**	**CD300a**	**CD300b**
Mus musculus	**Dna I2**	**KIF19**	**BTB(POZ) domain containing 17**	**GPR142**		**GPRC5C**	**CD300a**	**CD300b**
Rattus norvegicus	**Dna I2**	**KIF19**	**BTB(POZ) domain containing 17**	**GPR142**		**GPRC5C**	**CD300a**	**CD300b**
Bos taurus	**Dna I2**	**KIF19**	**BTB(POZ) domain containing 17**	**GPR142**		**GPRC5C**	**CD300a**	**CD300b**
Canis familiaris	**Dna I2**	**KIF19**	**BTB(POZ) domain containing 17**	**GPR142**		**GPRC5C**	**CD300a**	**CD300b**
Equus caballus	**Dna I2**	**KIF19**	**BTB(POZ) domain containing 17**	**GPR142**		**GPRC5C**	**CD300a**	**CD300b**
Gallus gallus	**Dna I2**	LOC771601 ψ	**BTB(POZ) domain containing 17**			**GPRC5C**	Ccdc137	C17orf90-like
Danio rerio	LOC564522 potassium channel	Map2k6	**BTB(POZ) domain containing 17**	**GPR142**	LOC100536759	**GPRC5C**	**CD300a**	**CD300b**

GPRC5B

Homo sapiens	**IQCK**	**GPRC5B**	**GPR139**	SALL4P3	small nuclear ribonucleoprotein polypeptide E ψ	**GP2**
Pan troglodytes	**IQCK**	**GPRC5B**	**GPR139**	SALL4P3	small nuclear ribonucleoprotein polypeptide E-like	**GP2**
Macaca Mulatta	**IQCK**	**GPRC5B**	**GPR139**	SALL4P3	small nuclear ribonucleoprotein polypeptide E-like	**GP2**
Callitrix jacchus	**IQCK**	**GPRC5B**	**GPR139**	SALL4P3		**GP2**
Mus musculus	**IQCK**	**GPRC5B**	**GPR139**		GM4080	**GP2**
Rattus norvegicus	**IQCK**	**GPRC5B**	**GPR139**			**GP2**
Bos taurus	**IQCK**	**GPRC5B**	**GPR139**			**GP2**
Canis familiaris	**IQCK**	**GPRC5B**	**GPR139**			**GP2**
Oryctolagus cuniculus	**IQCK**	**GPRC5B**	**GPR139**			**GP2**
Gallus gallus	**IQCK**	**GPRC5B**	**GPR139**	PDILT	ACSM5	ACSM4

GPRC5A and GPRC5D

Homo sapiens	**ApoL D1**	**DEAD box polypeptide 47**	Ribosomal protein L37a ψ	Ribosomal protein S6 ψ	ribosomal protein L13a ψ 20	**GPRC5A**	Mir614	**GPRC5D**		**HEBP 1**
Pan troglodytes	**ApoL D1**	**DEAD box polypeptide 47**	Ribosomal protein L37a ψ	Ribosomal protein S6 ψ	ribosomal protein L13a ψ 20	**GPRC5A**	Mir614	**GPRC5D**		**HEBP 1**
Macaca mulatta		**DEAD box polypeptide 47**	Ribosomal protein L37a ψ	Ribosomal protein S6 ψ		**GPRC5A**		**GPRC5D**		**HEBP1**
Mus musculus	**ApoL D1**	**DEAD box polypeptide 47**				**GPRC5A**		**GPRC5D**	similar to ribosomal prot. 17	**HEBP 1**
Rattus norvegicus	**ApoL D1**	**DEAD box polypeptide 47**				**GPRC5A**		**GPRC5D**	similar to ribosomal prot. 17	**HEBP 1**
Bos taurus	**ApoL D1**	**DEAD box polypeptide 47**				**GPRC5A**	eukaryotic translation initiation factor 3, ψ	**GPRC5D**		**HEBP 1**
Canis familiaris	**ApoL D1**	**DEAD box polypeptide 47**				**GPRC5A**		**GPRC5D**		**HEBP 1**
Gallus gallus	**ApoL D1**	**DEAD box polypeptide 47**				**GPRC5A**		**GPRC5D**		**HEBP 1**
