# Supplementary material for: Molecular evolution of a chordate specific family of G protein-coupled receptors
Source: BMC Evol Biol. 2011 Aug 9;11:234. doi: 10.1186/1471-2148-11-234 (PMC3238225; doi:10.1186/1471-2148-11-234)
Supplement: Additional file 6 — Sequence analysis of GPRC5 receptors. (A) Alignment of the first extracellular loops from different classes of GPRC5 receptors. (B) Putative IQ motifs in GPRC5 receptors. [file 1471-2148-11-234-S6.pdf]

A

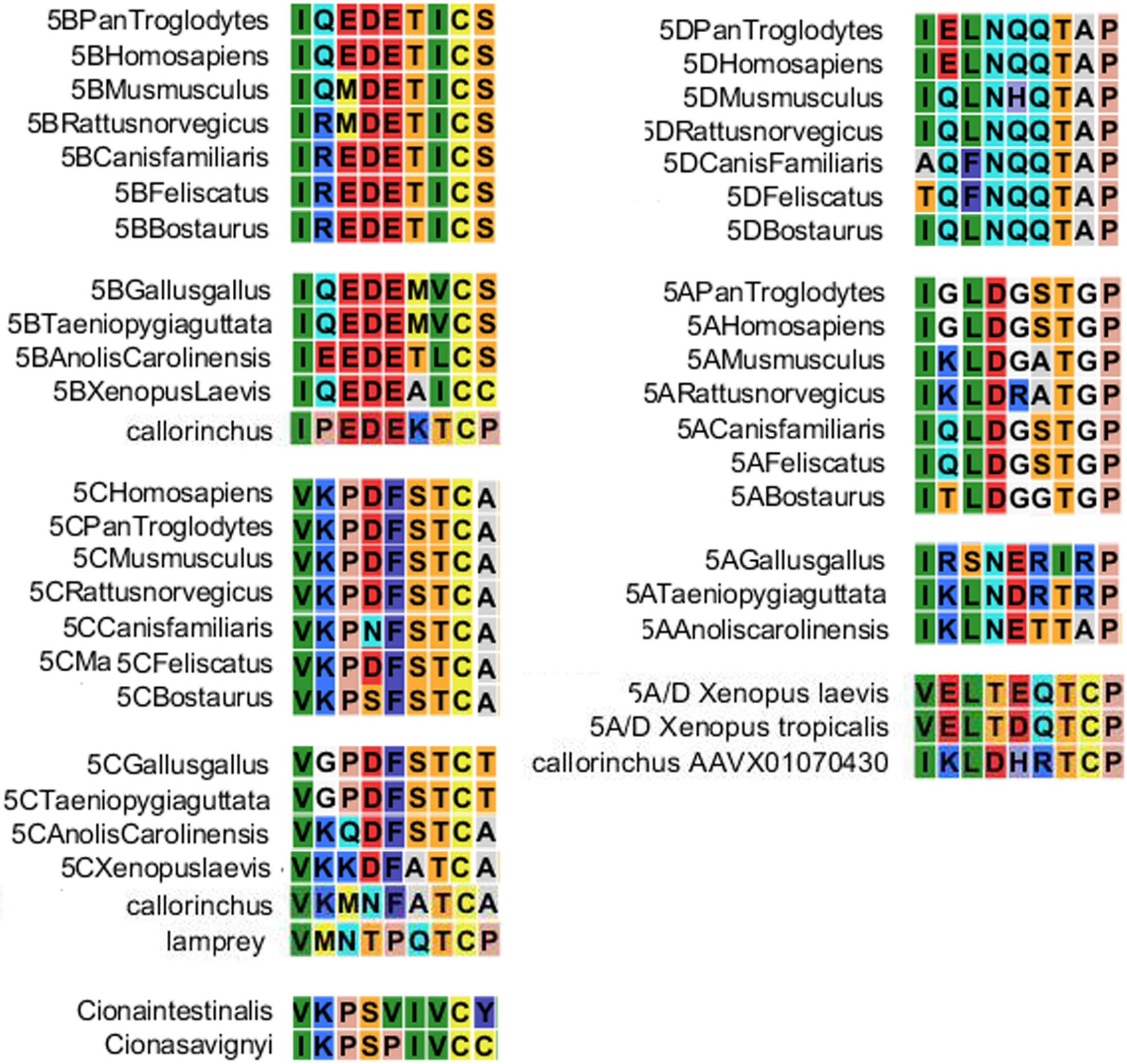

B

IL3 5C L C G R (YF) K R W R K H G (VI) F (VI) L L
1 5 8 14
1 5 10

IL3 5B L C G K F K R W K (LQ) N G (AV) (FC) (IL)(IL)(VI)
1 5 8 14
1 5 10

IL2 5A L L (AV) H A X X L X (RK) L (VA) R G X X P (LF) S
1 5 8 14
1 8 14
