# Supplementary material for: Molecular evolution of a chordate specific family of G protein-coupled receptors
Source: BMC Evol Biol. 2011 Aug 9;11:234. doi: 10.1186/1471-2148-11-234 (PMC3238225; doi:10.1186/1471-2148-11-234)
Supplement: Additional file 7 — Negative selection sites in GPRC5 receptors. GPRC5A and GPRC5D sequences from Mus musculus with marked sites where negative selection was predicted. [file 1471-2148-11-234-S7.pdf]

Mus musculus GPRC5A

Mttpraapsgcrsdldsryhrlcdlaegwgialetlaavgavatvacmfalvflickvqdsnkrkmlp

aqflfllgvlgvfgltfafiikldgatgptrfflfgvlfaicfscllahafnliklvrgrkplswlvi

lslavgfslvqdviaieylvltmnrtnvnvfselpaprrnedfvmlliyvlvlmvltffasflvfcgs

fsgwkrhgfhicftsflsiaiwvawivlllipdidrkwddtilstalvangwvflafyilpefrqlpr

qrsptdypvedafckpqlmkqsygvenraysqeeitqglemgdtlyapysthfqlqnhqkdfsipraq

apaspyndyegrkgds

Mus musculus GPRC5D

Myedcvkstedyylfcdnegpwaivleslavigivvtillllaflflmrkvqdcsqwnvlptqflfll

avlglfgltfafiiqlnhqtapvryflfgvlfaicfscllahasnlvklvrgrvsfcwttilfiaigv

sllqtiiaieyvtlimtrglmfehmtpyqlnvdfvclliyvlflmaltffvskatfcgpcenwkqhgr

lifatvlvsiiiwvvwismllrgnpqlqrqphwddaviciglvtnawvflliyiipelsilyrscrqe

cptqgnvcqvpvyqrsfrmdtqeptrec

Yellow – extracellular loops

Red – Sites showing negative selection (significance level p 0.1)
