# Supplementary material for: Molecular evolution of a chordate specific family of G protein-coupled receptors
Source: BMC Evol Biol. 2011 Aug 9;11:234. doi: 10.1186/1471-2148-11-234 (PMC3238225; doi:10.1186/1471-2148-11-234)
Supplement: Additional file 8 — mRNA sequences of Xenopus laevis sequences. Alignment of available mRNA sequences from XRAIG4 and GPRC5 receptors from Xenopus laevis. [file 1471-2148-11-234-S8.pdf]

| | | |
|---|---|---|
| | ATGACATCCAAACATTTCCTATTGATCCTG | |
| | 10 20 30 | |
| seq1(XRAIG4a)_EF456762.1.seq | ATGACATCCAAACATTTCCTATTGATCCTG | 30 |
| seq1(XRAIG4b)_EF456763.1.seq | ATGACATCCAGACATTTCCTACTGATCCTG | 30 |
| XRAIG4_NM_001173999.seq | ATGACATCCAAACATTTCCTATTGATCCTG | 30 |

| | | |
|---|---|---|
| | TGCATAATGGGCGCCATAAGGGACGCAGTG | |
| | 40 50 60 | |
| seq1(XRAIG4a)_EF456762.1.seq | TGCATAATGGGCGCCATAAGGGACGCAGTG | 60 |
| seq1(XRAIG4b)_EF456763.1.seq | TGCATGATGGGACCCATGAGAGAGGCATTG | 60 |
| XRAIG4_NM_001173999.seq | TGCATAATGGGCGCCATAAGGGACGCAGTG | 60 |

| | | |
|---|---|---|
| | TCTCTGAATCTCACAACTCCATCAGTGGAA | |
| | 70 80 90 | |
| seq1(XRAIG4a)_EF456762.1.seq | TCTCTGAATCTCACAACTCCATCAGTGGAA | 90 |
| seq1(XRAIG4b)_EF456763.1.seq | TCTCTGAATCTCACAACTCCATCAGTGGAA | 90 |
| XRAIG4_NM_001173999.seq | TCTCTGAATCTCACAACTCCATCAGTGGAA | 90 |

| | | |
|---|---|---|
| | TTTCAGAACCTCTCAGCTGTGCCCATGGGT | |
| | 100 110 120 | |
| seq1(XRAIG4a)_EF456762.1.seq | TTTCGGAACCTCTCAGCTGTGCCCATGGGT | 120 |
| seq1(XRAIG4b)_EF456763.1.seq | TTACAGAACCTCTCAGCTGTGCCCATGGGT | 120 |
| XRAIG4_NM_001173999.seq | TTTCAGAACCTCTCAGCTGTGCCCATGGGT | 120 |

| | | |
|---|---|---|
| | TGCAGTCGGGATGTCCCGTCCATTTTCTAC | |
| | 130 140 150 | |
| seq1(XRAIG4a)_EF456762.1.seq | TGCAGTCGGGATGTCCCGTCCATTTTCTAC | 150 |
| seq1(XRAIG4b)_EF456763.1.seq | TGCAGTCAGGATGTACCGTCAATATTCTAC | 150 |
| XRAIG4_NM_001173999.seq | TGCAGTCGGGATGTCCCGTCCATTTTCTAC | 150 |

| | | |
|---|---|---|
| | TTTCTGTGTGACCTGAATGCAGCGTGGGGC | |
| | 160 170 180 | |
| seq1(XRAIG4a)_EF456762.1.seq | TTTCTGTGTGACCTGAATGCAGCGTGGGGC | 180 |
| seq1(XRAIG4b)_EF456763.1.seq | TCTCTGTGTGACCTGAATGCAGCTTGGGGC | 180 |
| XRAIG4_NM_001173999.seq | TTTCTGTGTGACCTGAATGCAGCGTGGGGC | 180 |

| | | |
|---|---|---|
| | ATCATATTAGAGGCGTTGGCCGGGCTTGGA | |
| | 190 200 210 | |
| seq1(XRAIG4a)_EF456762.1.seq | ATCATATTAGAGGCGTTGGCCGGGCTTGGA | 210 |
| seq1(XRAIG4b)_EF456763.1.seq | ATTATATTAGAGGCGTTGGCCGGGCTTGGA | 210 |
| XRAIG4_NM_001173999.seq | ATCATATTAGAGGCGTTGGCCGGGCTTGGA | 210 |

| | | |
|---|---|---|
| Consensus | A T T A T C T G C T C T C T T C T A C T A G C T G T T A T C | 220 230 240 |
| seq1(XRAIG4a)_EF456762.1.seq | A T T A T C T G C T C T C T T C T A C T A G C T G T T A T C | 240 |
| seq1(XRAIG4b)_EF456763.1.seq | A T T A T C T G C T C T C T T C T A C T A G C T G T T A T C | 240 |
| XRAIG4_NM_001173999.seq | A T T A T C T G C T C T C T T C T A C T A G C T G T T A T C | 240 |

| | | |
|---|---|---|
| Consensus | T T C T T G G C A C T A G C A C C T T C G G G T A T G A G A | 250 260 270 |
| seq1(XRAIG4a)_EF456762.1.seq | T T C T T G G C A C T A G C A C C T T C G G G T A T G A G A | 270 |
| seq1(XRAIG4b)_EF456763.1.seq | T T C T T G G C A C T A G C A C C T T C G G G T A T G A G A | 270 |
| XRAIG4_NM_001173999.seq | T T C T T G G C A C T A G C A C C T T C G G T T A T G A G A | 270 |

| | | |
|---|---|---|
| Consensus | G A C G A G C G C A A A G G A T C C T T G G G C A T A A A C | 280 290 300 |
| seq1(XRAIG4a)_EF456762.1.seq | G A C G A G C G C A A A G G A T C C T T G G G C A T A A A C | 300 |
| seq1(XRAIG4b)_EF456763.1.seq | G A C G A G C G C A A A G G A T C C T T G G G C A T A A A C | 300 |
| XRAIG4_NM_001173999.seq | G A C G A G C G C A A A G G A T C C T T G G G C A T A A A C | 300 |

| | | |
|---|---|---|
| Consensus | T T T A T G T T C C T G T T T G G T G T C T T T G G C C T G | 310 320 330 |
| seq1(XRAIG4a)_EF456762.1.seq | T T T A T G T T C C T G T T T G G T G T C T T T G G C C T G | 330 |
| seq1(XRAIG4b)_EF456763.1.seq | T T T A T G T T C C T G T T T G G T G T C T T T G G C C T G | 330 |
| XRAIG4_NM_001173999.seq | T T T A T G T T C C T G T T T G G T G T C T T T G G C C T G | 330 |

| | | |
|---|---|---|
| Consensus | T T C T C T C T T G T T T T T G C C T T T A T C A T T G G A | 340 350 360 |
| seq1(XRAIG4a)_EF456762.1.seq | T T C T C T C T T G T T T T T G C C T T T A T C A T T G G A | 360 |
| seq1(XRAIG4b)_EF456763.1.seq | T T C T C T C T T G T T T T T G C C T T T A T C A T T G G A | 360 |
| XRAIG4_NM_001173999.seq | T T C T C T C T T G T T T T T G C C T T T A T C A T T G G A | 360 |

| | | |
|---|---|---|
| Consensus | C C G A A C A C G G C A G T G T G T G A A G T G A G A A G A | 370 380 390 |
| seq1(XRAIG4a)_EF456762.1.seq | C C G A A C A C G G C A G T G T G T G A A G T G A G A A G A | 390 |
| seq1(XRAIG4b)_EF456763.1.seq | C C G A A C A C G G C A G T G T G T G A A G T G A G A A G A | 390 |
| XRAIG4_NM_001173999.seq | C C G A A C A C G G C A G T G T G T G A A G T G A G A A G A | 390 |

| | | |
|---|---|---|
| Consensus | T T T C T A T T C G G A G T T T T G T T T G C C C T C T G C | 400 410 420 |
| seq1(XRAIG4a)_EF456762.1.seq | T T T C T A T T C G G A G T T T T G T T T G C C C T C T G C | 420 |
| seq1(XRAIG4b)_EF456763.1.seq | T T T C T A T T C G G A G T T T T G T T T G C C C T C T G C | 420 |
| XRAIG4_NM_001173999.seq | T T T C T A T T C G G A G T T T T G T T T G C C C T C T G C | 420 |

```
                                 T T T G C C A G C C T G G T G G C T C A C T C T G T G A G G
                                                   430                 440                 450
seq1(XRAIG4a)_EF456762.1.seq     T T T G C C A G C C T G G T G G C T C A C T C T G T G A G G  450
seq1(XRAIG4b)_EF456763.1.seq     T T T G C C A G C C T G G T G G C T C A C T C T G T G A G G  450
XRAIG4_NM_001173999.seq          T T T G C C A G C C T G G T G G C T C A C T C T G T G A G G  450

                                 C T G A A C T A C C T A G C A C T A C A C A A T C A C G G C
                                                   460                 470                 480
seq1(XRAIG4a)_EF456762.1.seq     C T G A A C T A C C T A G C A C T A C A C A A T C A C G G C  480
seq1(XRAIG4b)_EF456763.1.seq     C T G A A C T A C C T A G C A C T A C A C A A T C A C G G C  480
XRAIG4_NM_001173999.seq          C T G A A C T A C C T A G C A C T A C A C A A T C A C G G C  480

                                 C C A G G A G G A G G C T T G A T A T T T T T A C T C G C C
                                                   490                 500                 510
seq1(XRAIG4a)_EF456762.1.seq     C C A G G A G G A G G C T T G A T A T T T T T A C T C G C C  510
seq1(XRAIG4b)_EF456763.1.seq     C C A G G A G G A G G C T T G A T A T T T T T A C T C G C C  510
XRAIG4_NM_001173999.seq          C C A G G A G G A G G C T T G A T A T T T T T A C T C G C C  510

                                 A C T G G A C T T T T C T T G G T G G A G G C T G T A A T A
                                                   520                 530                 540
seq1(XRAIG4a)_EF456762.1.seq     A C T G G A C T T T T C T T G G T G G A G G C T G T A A T A  540
seq1(XRAIG4b)_EF456763.1.seq     A C T G G A C T T T T C T T G G T G G A G G C T G T A A T A  540
XRAIG4_NM_001173999.seq          A C T G G A C T T T T C T T G G T G G A G G C T G T A A T A  540

                                 A A T G C A G A A T G G C T T C T C A T C A C T A A T G T C
                                                   550                 560                 570
seq1(XRAIG4a)_EF456762.1.seq     A A T G C A G A A T G G C T T C T C A T C A C T A A T G T C  570
seq1(XRAIG4b)_EF456763.1.seq     A A T G C A G A A T G G C T T C T C A T C A C T A A T G T C  570
XRAIG4_NM_001173999.seq          A A T G C A G A A T G G C T T C T T A T C A C T A A T G T C  570

                                 C G A C A T A A C C T T C C A T C A G C T A T C T C A A T A
                                                   580                 590                 600
seq1(XRAIG4a)_EF456762.1.seq     C G A C A T A A C C T T C C A T C A G C T A T C T C A A T A  600
seq1(XRAIG4b)_EF456763.1.seq     C G A C A T A A C C T T C C A T C A G C T A T C T C A A T A  600
XRAIG4_NM_001173999.seq          C G A C A T A A C C T T C C A T C A G C T A T C T C A A T A  600

                                 G G A C A T C C T T G T A A T A T A G A T A A C C A A G A C
                                                   610                 620                 630
seq1(XRAIG4a)_EF456762.1.seq     G G A C A T C C T T G T A A T A T A G A T A A C C A A G A C  630
seq1(XRAIG4b)_EF456763.1.seq     G G A C A T C C T T G T A A T A T A G A T A A C C A A G A C  630
XRAIG4_NM_001173999.seq          G G A C A T C C T T G T A A T A T A G A T A A C C A A G A C  630
```

```
                                T T T G T G A C A T C T T T A G T C T A T G T C A T G T T T
                                                  640                 650                 660
seq1(XRAIG4a)_EF456762.1.seq    T T T G T G A C A T C T T T A G T C T A T G T C A T G T T T   660
seq1(XRAIG4b)_EF456763.1.seq    T T T G T G A C A T C T T T A G T C T A T G T C A T G T T T   660
XRAIG4_NM_001173999.seq         T T T G T G A C A T C T T T A G T C T A T G T C A T G T T T   660

                                C T T A T T C T A G C C G C T C T G A T T A T T C C T T G C
                                                  670                 680                 690
seq1(XRAIG4a)_EF456762.1.seq    C T T A T T C T A G C C G C T C T G A T T A T T C C T T G C   690
seq1(XRAIG4b)_EF456763.1.seq    C T T A T T C T A G C C G C T C T G A T T A T T C C T T G C   690
XRAIG4_NM_001173999.seq         C T T A T T C T A G C C G C T C T G A T T A T T C C T T G C   690

                                C C G G T A C T T T G C G G A C A C T A T X X X X X X X X X
                                                  700                 710                 720
seq1(XRAIG4a)_EF456762.1.seq    C C G G T A C T T T G C G G A C A C T A T                     711
seq1(XRAIG4b)_EF456763.1.seq    C C G G T A C T T T G C G G A C A C T A T                     711
XRAIG4_NM_001173999.seq         C C G G T A C T T T G C G G A C A C T A T T T G C C T T G G   720

                                X X X X X X X X X X X X X X X X X X X X X X X X X X X X X X
                                                  730                 740                 750
seq1(XRAIG4a)_EF456762.1.seq                                                                  711
seq1(XRAIG4b)_EF456763.1.seq                                                                  711
XRAIG4_NM_001173999.seq         A A A C G C C A T G G C A A G T A T A T T G T G G T A A C T   750

                                X X X X X X X X X X X X X X X X X X X X X X X X X X X X X X
                                                  760                 770                 780
seq1(XRAIG4a)_EF456762.1.seq                                                                  711
seq1(XRAIG4b)_EF456763.1.seq                                                                  711
XRAIG4_NM_001173999.seq         G C C T T C C T C T C T C T T T C C A T C T G G G T G A T C   780

                                X X X X X X X X X X X X X X X X X X X X X X X X X X X X X X
                                                  790                 800                 810
seq1(XRAIG4a)_EF456762.1.seq                                                                  711
seq1(XRAIG4b)_EF456763.1.seq                                                                  711
XRAIG4_NM_001173999.seq         T G G A T T G T G A T G T A C C T C T A T G G C A A T G A C   810

                                X X X X X X X X X X X X X X X X X X X X X X X X X X X X X X
                                                  820                 830                 840
seq1(XRAIG4a)_EF456762.1.seq                                                                  711
seq1(XRAIG4b)_EF456763.1.seq                                                                  711
XRAIG4_NM_001173999.seq         A A A T T A G G C C A G C G G T A C T G G G A T G A C C C A   840
```

```
                                 XXXXXXXXXXXXXXXXXXXXXXXXXXXXXX
                                          850       860       870
seq1(XRAIG4a)_EF456762.1.seq                                         711
seq1(XRAIG4b)_EF456763.1.seq                                         711
XRAIG4_NM_001173999.seq          GTTCTTGCCATTGCTTTAGTGTCCAATGGC      870

                                 XXXXXXXXXXXXXXXXXXXXXXXXXXXXXX
                                          880       890       900
seq1(XRAIG4a)_EF456762.1.seq                                         711
seq1(XRAIG4b)_EF456763.1.seq                                         711
XRAIG4_NM_001173999.seq          TGGGTATTTATTTTCTTTTACTTTATTCCG      900

                                 XXXXXXXXXXXXXXXXXXXXXXXXXXXXXX
                                          910       920       930
seq1(XRAIG4a)_EF456762.1.seq                                         711
seq1(XRAIG4b)_EF456763.1.seq                                         711
XRAIG4_NM_001173999.seq          GAACTCCTGGAAATGACAAGGACGGGGTAT      930

                                 XXXXXXXXXXXXXXXXXXXXXXXXXXXXXX
                                          940       950       960
seq1(XRAIG4a)_EF456762.1.seq                                         711
seq1(XRAIG4b)_EF456763.1.seq                                         711
XRAIG4_NM_001173999.seq          GGATACGAGACAGAAACCCTGAACATGATA      960

                                 XXXXXXXXXXXXXXXXXXXXXXXXXXXXXX
                                          970       980       990
seq1(XRAIG4a)_EF456762.1.seq                                         711
seq1(XRAIG4b)_EF456763.1.seq                                         711
XRAIG4_NM_001173999.seq          AAAAGGTTTGAGGAACGTCCACCTTCTTTC      990

                                 XXXXXXXXXXXXXXXXXXXXXXXXXXXXXX
                                         1000      1010      1020
seq1(XRAIG4a)_EF456762.1.seq                                         711
seq1(XRAIG4b)_EF456763.1.seq                                         711
XRAIG4_NM_001173999.seq          ATTATGGAGAACAGGGCGTTCACTATGGAA      1020

                                 XXXXXXXXXXXXXXXXXXXXXXXXXXXXXX
                                         1030      1040      1050
seq1(XRAIG4a)_EF456762.1.seq                                         711
seq1(XRAIG4b)_EF456763.1.seq                                         711
XRAIG4_NM_001173999.seq          AACCTGGAGATGACTGATCACGTGGAAACT      1050
```

| | | |
|---|---|---|
| | X X X X X X X X X X X X X X X X X X X X X X X X X X X X X X | |
| | 1060 1070 1080 | |
| seq1(XRAIG4a)_EF456762.1.seq | | 711 |
| seq1(XRAIG4b)_EF456763.1.seq | | 711 |
| XRAIG4_NM_001173999.seq | A T A A A A A T C C A A G A C A A A C C T G T T T C T C C G | 1080 |

| | | |
|---|---|---|
| | X X X X X X X X X X X X X X X X X X X X X X X X X X X X X X | |
| | 1090 1100 1110 | |
| seq1(XRAIG4a)_EF456762.1.seq | | 711 |
| seq1(XRAIG4b)_EF456763.1.seq | | 711 |
| XRAIG4_NM_001173999.seq | T A C A G T A A C T A T T G C G G A C T C T A T C C A A C G | 1110 |

| | | |
|---|---|---|
| | X X X X X X X X X X X X X X X X X X X X X X X X X X X X X X | |
| | 1120 1130 1140 | |
| seq1(XRAIG4a)_EF456762.1.seq | | 711 |
| seq1(XRAIG4b)_EF456763.1.seq | | 711 |
| XRAIG4_NM_001173999.seq | T T G C C T T T G T A C C C A T C G G A T G T G G A G A C A | 1140 |

| | | |
|---|---|---|
| | X X X X X X X X X X X X X X X X X X X X X X X X X X X X X X | |
| | 1150 1160 1170 | |
| seq1(XRAIG4a)_EF456762.1.seq | | 711 |
| seq1(XRAIG4b)_EF456763.1.seq | | 711 |
| XRAIG4_NM_001173999.seq | G T C A A C C A C G T A C C T C T G C C G C G C A T T T C C | 1170 |

| | | |
|---|---|---|
| | X X X X X X X X X X X X X X X X X X X X X X X X X X X | |
| | 1180 1190 | |
| seq1(XRAIG4a)_EF456762.1.seq | | 711 |
| seq1(XRAIG4b)_EF456763.1.seq | | 711 |
| XRAIG4_NM_001173999.seq | A T G G A G C C C T G G A G A T A C C G T C T G T A G | 1197 |
