# Supplementary material for: Molecular evolution of a chordate specific family of G protein-coupled receptors
Source: BMC Evol Biol. 2011 Aug 9;11:234. doi: 10.1186/1471-2148-11-234 (PMC3238225; doi:10.1186/1471-2148-11-234)
Supplement: Additional file 9 — GPRC5D from dolphin. Alignment of GPRC5D from Tursiops truncatus with the consensus of all mammalian GPRC5D sequences. [file 1471-2148-11-234-S9.pdf]

```
                                                                     
5D Tursiops truncatus  MNEDCEESTG DCYFLCDTVG AWGIVLES-A SIGLAVTVLP P-AFLFLMRR  48
Consensus 5D mammals   MYEDCIESTG D-YYLCDTEG PWGIVLESLA IIGIVVTILL LLAFLFLMRK  49

                               60                    80                   100
                                |                     |                     |
5D Tursiops truncatus  VQDRSQWNVL PTQFLFLLGG LGLLSLAFAF LIQLNQQTAP LHYFLFGVLF  98
Consensus 5D mammals   VQDCSQWNVL PTQFLFLLGV LGLFGLAFAF IIQLNQQTAP VRYFLFGVLF  99

5D Tursiops truncatus  AVCFSCLLTH ASNLVELVWG QVSSSWTTIL CTAIGVSLWQ TIIATEYVTL 148
Consensus 5D mammals   ALCFSCLLAH ASNLVKLVRG RVSFSWTTIL CIAIGCSLLQ TIIAIEYVTL 149

                                                    180
                                                      |
5D Tursiops truncatus  IVTRGTMFMD MTPCQLNVDF AVLLVYVLFL LALTFFVSKA TFCGPCEDWK 198
Consensus 5D mammals   IMTRGMMFVH MTPCQLNVDF VVLLVYVLFL MALTFFVSKA TFCGPCENWK 199

                                          220                   240
                                            |                     |
5D Tursiops truncatus  RRGRLVFLTA LISIIIWVVW ISMLMRG--- -----QWDDP ILCIALVTHA 240
Consensus 5D mammals   QHGRLIFVTV LISIIIWVVW ISMLLRGNPQ LQRQPQWDDP VICIALVTNA 249

                               260                   280                   300
                                |                     |                     |
5D Tursiops truncatus  WVFLLLYIIP ELCFL-RSCQ QDCPLQGNTC PLPA-ECSFR VENQQLSR-- 286
Consensus 5D mammals   WVFLLLYIIP ELCILYRSCR QDCPLQGNAC PVPAYQRSFR VENQELSRAR 299

                                          320                   340
                                            |                     |
5D Tursiops truncatus  ---------- ---------- -TVDPTQEDF IPWAKVSPQQ DAEL 309
Consensus 5D mammals   DSDGAEEDVA LTSYGTPIQL QTVDPTQEYF IPRAKLSPQQ DAG- 342
```
