# Supplementary material for: Molecular evolution of a chordate specific family of G protein-coupled receptors
Source: BMC Evol Biol. 2011 Aug 9;11:234. doi: 10.1186/1471-2148-11-234 (PMC3238225; doi:10.1186/1471-2148-11-234)
Supplement: Additional file 12 — Additional Materials. Figure legends of additional files, list of accession numbers of sequences included in this study, qPCR information. [file 1471-2148-11-234-S12.doc]

**Additional files / Figure Legends.**

**Additional file 1. Phylogenetic analysis.** (**A**) Phylogenetic tree obtained by RAxML, branch labels indicate bootstrap values. Branch lengths represent phylogenetic distances. (**B**) Screening of XRAIG4 expression in different tissues from *Xenopus laevis* by RT-PCR, ODC serves as a positive control.

**Additional file 2**. Alignment showing a sequence between the end of the N-terminus and the transition into transmembrane region 1 from different GPRC5 receptors.

**Additional file 3**. Genes in the vicinity of GPRC5 receptors from different species. GPRC5 genes are marked in red, genes present in the majority of species are bold (ψ = pseudogene).

**Additional file 4**. (**A)** Sequence logos of all GPRC5 receptor sequences found in mammals. Polar amino acids in green, basic in blue, acidic in red and hydrophobic in black, the seven transmembrane regions (TM1-7) are indicated with black lines. **(A)** GPRC5A **(B)** GPRC5B **(C)** GPRC5C **(D)** GPRC5D.

**Additional file 5**. Evolutionary conservation of GPRC5 receptors. Hydropathy plots (black) and degree of conservation (red) of GPRC5 consensus (1 = 100%) sequences from selected representatives of different species. (**A**) GPRC5A sequences from tetrapods (human, chicken, frog). (**B**) GPRC5B sequences from chordates (human, chicken, zebrafish, frog). (**C**) GPRC5C sequences from chordates. (human, chicken, zebrafish, frog) (**D**) Plot for the consensus sequence derived from all receptor sequences.

**Additional file 6**. (**A)** Alignment of the first extracellular loops from different classes of GPRC5 receptors. Unique sequence motives, which are conserved between species, can be seen for each member. (**B)** Putative IQ motifs in GPRC5 receptors.

**Additional file 7**. GPRC5A and GPRC5D sequences from *Mus musculus*. Extracellular domains are marked in yellow, site where negative selection was predicted are marked in red.

**Additional file 8**. Alignment of GPRC5D from *Tursiops truncatus* with the consensus of all mammalian GPRC5D sequences. Arrows indicate amino acid changes that are putatively important for protein function are indicated by arrows. Especially the introduction of two proline residues at the end of TM1, which can lead to changes in the conformation of this part of the protein by inducing hinges, and the replacement of three positively charged amino acids in IL2 by negatively charged or uncharged residues can have implications on the function of the protein and might be linked to the loss of hair in this species.

**Additional file 9**. Alignment of available mRNA sequences from XRAIG4 and GPRC5 receptors from *Xenopus laevis*. XRAIG4a and XRAIG4b were classified as pseudoalleles due to gene duplication in the *Xenopus laevis* lineage[16], but could also represent splice variants. Since the differences are only located in a short stretch of amino acids at the N-terminus, they also may represent splice variants or polymorphisms from the same gene, or even sequencing errors. Moreover, in support of this hypothesis, also the nucleotide sequences show changes only at the position that lead to amino acid changes at the n-terminus, no changes were found at the c-terminal part of the sequence. Both sequences are incomplete, but a complete sequence (termed here XRAIG4) can be found in the database which is identical to XRAIG4a at the N-terminus. Taken together, we think that only one XRAIG4 gene exists in *Xenopus laevis* (as in *Xenopus tropicalis*).

**Additional Material**

**Accession numbers of sequences used in this study**

5B Danio Rerio XP_698748

5B-2 Danio Rerio EMBL CAX13908.1

5C Danio Rerio EMBL AAI5194.1

5Ba Tetraodon nigroviridis CAG11409

5Bb Tetraodon nigroviridis CAG06055

5Ca Tetraodon nigroviridis CAG03050

5Cb Tetraodon nigroviridis CAG00236

5Ba Gasterosteus aculeatus ENSGACP00000015129

5Bb Gasterosteus aculeatus ENSGACP00000025040

5Ca Gasterosteus aculeatus ENSGACP00000019048

5Cb Gasterosteus aculeatus ENSGACP00000014443

5A/D Gasterosteus aculeatus ENSGACP00000009488

5Ba Takifugu rubripes ENSTRUP00000040704

5Bb Takifugu rubripes ENSTRUP00000019362

5Ca Takifugu rubripes ENSTRUP00000005434

5Cb Takifugu rubripes ENSTRUP00000046163

5A/D Takifugu rubripes ENSTRUP00000019761

5Ba Oryzias latipes ENSORLP00000010721

5Bb Oryzias latipes ENSORLP00000014578

5Ca Oryzias latipes ENSORLP00000017304

5Cb Oryzias latipes ENSORLP00000009425

5A/D Oryzias latipes Sequence was found on Chromosome 8 20312691- 20313443

5A Rattus norvegicus ABBA01074152

5B Rattus norvegicus NP_001099774

5C Rattus norvegicus AAI05782

5D Rattus norvegicus XP_001070147

5A Mus musculus EDL10540

5B Mus musculus EDL17154

5C Mus musculus CAM18754

5D Mus musculus Q7JIL6

5A Tupaia belangeri ENSTBEP00000011779

5B Tupaia belangeri ENSTBEP00000000413

5C Tupaia belangeri ENSTBEP00000004373

5D Tupaia belangeri ENSTBEP00000011939

5A Cavia porcellus ENSCPOP00000009564

5B Cavia porcellus ENSCPOP00000002187

5C Cavia porcellus ENSCPOP00000020458

5D Cavia porcellus ENSCPOP00000017442

5A Monodelphis domestica ENSMODP00000022718

5B Monodelphis domestica ENSMODP00000008222

5C Monodelphis domestica ENSMODP00000008442

5D Monodelphis domestica ENSMODP00000022717

5A Ochotona princeps ENSOPRP00000013195

5B Ochotona princeps ENSOPRP00000002138

5C Ochotona princeps ENSOPRP00000012647

5D Ochotona princeps ENSOPRP00000013205

5A Oryctolagus cuniculus ENSOCUP00000014225

5B Oryctolagus cuniculus ENSOCUP00000001656

5C Oryctolagus cuniculus ENSOCUP00000011534

5D Oryctolagus cuniculus ENSOCUP00000013174

5A Sorex araneus ENSSARP00000005437

5C Sorex araneus ENSSARP00000006735

5D Sorex araneus ENSSARP00000000620

5A Spermophilus tridecemlineatus ENSSTOP00000001654

5B Spermophilus tridecemlineatus ENSSTOP00000013633

5D Spermophilus tridecemlineatus ENSSTOP00000000555

5B Dipodomys ordii ENSDORP00000000764

5C Dipodomys ordii ENSDORP00000005066

5D Dipodomys ordii ENSDORP00000000464

5A Homo sapiens EAW96289

5B Homo sapiens EAW50302

5C Homo sapiens NP_071319

5D Homo sapiens AAH69341

5A Pan troglodytes XP_001154537

5B Pan troglodytes XP_001154664

5C Pan troglodytes XP_001154938

5D Pan troglodytes ENSPTRP00000059501

5A Macaca mulatta XP_001086465

5B Macaca mulatta XP_001084286

5C Macaca mulatta XP_001087385

5D Macaca mulatta ENSMMUP00000040185

5A Gorilla gorilla ENSGGOP00000015530

5C Gorilla gorilla ENSGGOP00000002960

5D Gorilla gorilla ENSGGOP00000000859

5B Pongo pygmaeus ENSPPYP00000008087

5C Pongo pygmaeus ENSPPYP00000009654

5D Pongo pygmaeus ENSPPYP00000004924

5A Microcebus murinus ENSMICP00000009193

5B Microcebus murinus ENSMICP00000010128

5C Microcebus murinus ENSMICP00000007797

5D Microcebus murinus ENSMICP00000009196

5A Otolemur garnettii ENSOGAP00000004308

5B Otolemur garnettii ENSOGAP00000008999

5C Otolemur garnettii ENSOGAP00000005974

5D Otolemur garnettii ENSOGAP00000009079

5B Tarsius syrichta ENSTSYP00000009473

5D Tarsius syrichta ENSTSYP00000010839

5A Bos taurus XP_611599

5B Bos taurus AAI49137

5C Bos taurus AAI10242

5D Bos taurus XP_614909

5A Canis familiaris XP_543807

5B Canis familiaris XP_536950

5C Canis familiaris ENSCAFP00000006790

5D Canis familiaris ENSCAFP00000035696

5A Tursiops truncatus ENSTTRP00000012149

5B Tursiops truncatus ENSTTRP00000004750

5C Tursiops truncatus ENSTTRP00000013285

5D Tursiops truncatus ENSTTRP00000012153

5A Equus caballus ENSECAP00000012545

5B Equus caballus ENSECAP00000019959

5C Equus caballus ENSECAP00000014701

5D Equus caballus ENSECAP00000013821

5A Felis catus ENSFCAP00000012974

5B Felis catus ENSFCAP00000012974

5C Felis catus ENSFCAP00000008098

5D Felis catus ENSFCAP00000012975

5A Pteropus vampyrus ENSPVAP00000014060

5B Pteropus vampyrus ENSPVAP00000002560

5C Pteropus vampyrus ENSPVAP00000016481

5D Pteropus vampyrus ENSPVAP00000000286

5A Loxodonta africana ENSLAFP00000008559

5B Loxodonta africana ENSLAFP00000016845

5C Loxodonta africana ENSLAFP00000008975

5D Loxodonta africana ENSLAFP00000012976

5A Myotis lucifugus ENSMLUP00000002880

5B Myotis lucifugus ENSMLUP00000007654

5C Myotis lucifugus ENSMLUP00000011288

5D Myotis lucifugus ENSMLUP00000002887

5A Dasypus novemcinctus ENSDNOP00000014636

5C Dasypus novemcinctus ENSDNOP00000007604

5D Dasypus novemcinctus ENSDNOP00000015544

5A Echinops telfairi ENSETEP00000008081

5B Echinops telfairi ENSETEP00000016359

5C Echinops telfairi ENSETEP00000016209

5D Echinops telfairi ENSETEP00000010705

5A Erinaceus europaeus ENSEEUP00000005246

5B Erinaceus europaeus ENSEEUP00000008509

5C Erinaceus europaeus ENSEEUP00000003838

5D Erinaceus europaeus ENSECAP00000013821

5A Procavia capensis ENSPCAP00000007535

5B Procavia capensis ENSPCAP00000006203

5C Procavia capensis ENSPCAP00000001058

5D Procavia capensis ENSPCAP00000007544

5D Choloepus hoffmanni ENSCHOP00000000099

5D Vicugna pacos ENSVPAP00000004314

5A Macropus eugenii ENSMEUP00000014892

5B Macropus eugenii ENSMEUP00000011775

5C Macropus eugenii ENSMEUP00000009471

5D Macropus eugenii ENSMEUP00000014900

5A Ornithorhynchus anatinus XP_001521580

5B Ornithorhynchus anatinus XP_001508999

5C Ornithorhynchus anatinus ENSOANP00000005212

5D Ornithorhynchus anatinus ENSOANP00000010517

5A Gallus gallus XP_416200

5B Gallus gallus XP_001233984

5C Gallus gallus XP_425386

5A Taeniopygia guttata XP_002191560

5B Taeniopygia guttata XP_002193116

5C Taeniopygia guttata XP_002191926

5A Anolis carolinensis ENSACAP00000010441

5B Anolis carolinensis ENSACAP00000002632

5C Anolis carolinensis ENSACAP00000014805

5A Xenopus laevis NP_001083364

5B Xenopus laevis NP_001093384

5C Xenopus laevis NP_001093385

XRaig4 Xenopus laevis AAI55366

5A Xenopus tropicalis Sequence was found on [http://genome.jgi-psf.org/](http://genome.jgi-psf.org/Xentr4/Xentr4.home.html)

Protein ID = 293010

5B Xenopus tropicalis Sequence was found on [http://genome.jgi-psf.org/](http://genome.jgi-psf.org/Xentr4/Xentr4.home.html)

Protein ID = 309156

5C Xenopus tropicalis NP_001011349

XRaig4 Xenopus tropicalis Sequence was found on [http://genome.jgi-psf.org/](http://genome.jgi-psf.org/Xentr4/Xentr4.home.html)

Protein ID = 298117

5C Callorinchus millii ti|1573943182

5B Callorinchus milii translated from AAVX01555109.1

5A/D Callorinchus milii translated from AAVX01070430.1

5 Ciona intestinalis XP_002129397

5 Ciona savignyii ENSCSAVP00000015339

5 Petromyzon marinus Sequence was found on [http://genome.ucsc.edu](http://genome.ucsc.edu/), Contig20850:1187-2338

5 Petromyzon marinus Sequence was found on [http://genome.ucsc.edu](http://genome.ucsc.edu/), Contig67271:853-2262

5 Branchiostoma floridae XP_002605967

5B Caenorhabditis elegans NP_501400

5B Caenorhabditis briggsae XP_002634053

BOSS Drosophila melanogaster NP_542440

HS_mGluR7 NP_870989

HS_mGluR8 NP_000836

HS_mGluR1 AAI36281

HS_mGluR1 isoform NP_000829

HS_mGluR1 beta NP_001107801

MM_V2R2 AAC08413

HS_CASR AAI12237

HS_GPRC6a AAM22230

HS_GABAB(1c) CAA09941

HS_GABAB(1a) CAA09939

HS_GABAB(2) CAA09942

Nearest Hit Dictostelium discoideum XP_645481

Boss-like Anopheles gambiae XP_313193

Additional accession numbers of sequences used in figure 4

5B Mus musculus TV1 NP_001182703

5B Mus musculus TV2 NP_071865

5C Homo sapiens TV1 NP_071319

5C Homo sapiens TV2 NP_061123

5C Mus musculus TV1 NP_001103807

5C Mus musculus TV2/3 NP_001103808

5D Mus musculus TV1 NP_001192325

5D Mus musculus TV2 NP_444348

5 Petromyzon marinus GENSCAN00000114119, Contig 20850: 1,187-2,339
